# The Influence of Exogenous Jasmonic Acid on the Biosynthesis of Steroids and Triterpenoids in *Calendula officinalis* Plants and Hairy Root Culture

**DOI:** 10.3390/ijms232012173

**Published:** 2022-10-12

**Authors:** Agata Rogowska, Małgorzata Stpiczyńska, Cezary Pączkowski, Anna Szakiel

**Affiliations:** 1 Department of Plant Biochemistry, Faculty of Biology, University of Warsaw, 1 Miecznikowa Street, 02-096 Warsaw, Poland; 2 Botanic Garden, Faculty of Biology, University of Warsaw, 4 Al. Ujazdowskie Street, 00-478 Warsaw, Poland

**Keywords:** elicitation, jasmonic acid, metabolic modifications, sterols, specialized metabolites, oleanolic acid, triterpenoids

## Abstract

The interplay between steroids and triterpenoids, compounds sharing the same biosynthetic pathway but exerting distinctive functions, is an important part of the defense strategy of plants, and includes metabolic modifications triggered by stress hormones such as jasmonic acid. Two experimental models, *Calendula officinalis* hairy root cultures and greenhouse cultivated plants (pot plants), were applied for the investigation of the effects of exogenously applied jasmonic acid on the biosynthesis and accumulation of steroids and triterpenoids, characterized by targeted GC-MS (gas chromatography-mass spectroscopy) metabolomic profiling. Jasmonic acid elicitation strongly increased triterpenoid saponin production in hairy root cultures (up to 86-fold) and their release to the medium (up to 533-fold), whereas the effect observed in pot plants was less remarkable (two-fold enhancement of saponin biosynthesis after a single foliar application). In both models, the increase of triterpenoid biosynthesis was coupled with hampering the biomass formation and modifying the sterol content, involving stigmasterol-to-sitosterol ratio, and the proportions between ester and glycoside conjugates. The study revealed that various organs in the same plant can react differently to jasmonic acid elicitation; hairy root cultures are a useful in vitro model to track metabolic changes, and enhanced glycosylation (of both triterpenoids and sterols) seems to be important strategy in plant defense response.

## 1. Introduction

Plants’ acclimation to environmental changes, as well as defense strategies against biotic and abiotic stress factors, involve a complex metabolic remodeling triggered by signaling molecules such as jasmonic acid [1,2]. It is generally postulated that the reallocation of energy and metabolic resources drives the antagonism between plant growth and defense, resulting in pathway competition between general and specialized metabolism [3,4]. In the context of the growth-defense trade-off, the course of metabolic modifications of steroids and triterpenoids is particularly intriguing. 

Steroids and triterpenoids are two groups of isoprenoids produced by folding and cyclization of their common precursor squalene. These compounds are commonly considered general and specialized metabolites based on their distinctive functions; that is, the membrane architectural and regulatory role of sterols versus the participation of triterpenoids in plant chemical protection and interactions with the environment [4,5]. This traditional classification has frequently been questioned since the border between general and specialized metabolism is not always precise and easy to demarcate in specific conditions. However, regardless of this debatable nomenclature [6], steroids and triterpenoids share the same biosynthetic precursor pathway—their interplay is therefore undoubtedly the important part of the metabolic strategy of plants under stress.

Steroids and triterpenoids occur in significant amounts in *Calendula officinalis* L., a plant which was chosen as an experimental model for the present study. *C. officinalis* (known as marigold) is an annual herb in the Asteraceae family, and although it is valued for its ornamental properties, it is mainly cultivated as a medicinal plant for use in the pharmaceutical and cosmetic industries [7]. *C. officinalis* extracts were traditionally used in the treatment of many skin disorders, such as wounds, tumors, dermatological lesions, ulcers, swellings, and exfoliative cheilitis, but also for gastro-intestinal inflammations and in dysmenorrhea [8]. *C. officinalis* is particularly rich in triterpenoid glycosides (saponins) with an oleanane core, which are considered to be responsible for some of the beneficial activities associated with pharmaceutical and cosmetic formulations [9]. To obtain a high triterpene saponin yield, an in vitro hairy root culture model of *C. officinalis* was previously designed using the wild-type *Agrobacterium rhizogenes* strain ATCC 15834 [10]. Hairy root in vitro cultures constitute a convenient method to increase the production efficiency of desired compounds by using bioreactor cultivation and elicitation with various elicitors [4,10]. An additional benefit of *C. officinalis* hairy root cultures is that oleanane saponins not only accumulate within the culture biomass, but are mainly released into the medium itself, which greatly facilitates their extraction. Previous studies on the enhancement of saponin productivity in *C. officinalis* hairy root cultures have pointed to jasmonic acid as the most effective of the applied elicitors [11]. Indeed, jasmonic acid is considered to be an effective elicitor in biotechnology (including various plant in vitro culture types) and is also applied as an inductor of natural plant resistance in modern sustainable agriculture [12].

Jasmonic acid and its analogues, methyl jasmonate and jasmonoyl isoleucine, belong to a phytohormone group called jasmonates, and regulate many distinct physiological plant responses [13,14]. Jasmonates, together with such hormones as salicylic acid, or ethylene, participate in plant defense mechanism activation, and are believed to be key regulators in acquiring resistance against various biotic and abiotic stresses [1,2]. Studies performed on numerous plant species have confirmed the role of jasmonates in inducing the biosynthesis of various specialized plant metabolite classes [13,15]. Therefore, jasmonate elicitation is now one of the most effective strategies for obtaining a high yield of specialized metabolites, both in plant in vitro cultures as well as in cultivated medicinal and crop plants [16,17,18]. The exogenous application of jasmonic acid, or its derivatives, is commonly used for enhancing the production of economically useful and specialized metabolites such as taxanes, morphine, or ginsenosides [19,20,21]. 

Although jasmonate elicitation has been considered particularly effective for boosting the biosynthesis of specialized metabolites, it has often been observed that this positive effect hampers biomass formation [18,22]. This phenomenon is often explained as a result of growth-defense trade-off, involving competition between various metabolic pathways, e.g., sterol and triterpenoid biosynthesis. Sterol-triterpenoid competition was originally observed in studies on plants, or plant in vitro cultures, producing either free form triterpenoids or saponins [4,11]. For example, methyl jasmonate application enhanced the production of saikosaponins (triterpenoid oleanane-type saponins) in wild-type adventitious roots of medicinal plant *Bupleurum falcatum,* but suppressed sterol biosynthesis [23]. Likewise, centelloside (triterpenoid ursane-type saponins) content in hairy roots of *Centella asiatica* increased after methyl jasmonate treatment, while the sterol content simultaneously decreased [24]. It was demonstrated that the increased production of ginsenosides in *Panax notoginseng* adventitious root culture after elicitation with jasmonic acid was coupled with upregulation of the expression of geranyl diphosphate synthase, farnesyl diphosphate synthase, squalene synthase, squalene epoxidase, dammarenediol synthase; and simultaneously with downregulation of the expression of cycloartenol synthase, involved in sterol biosynthesis [25].

Numerous reports on the successful application of jasmonates to enhance the production of pharmacologically important saponins, e.g., glycyrrhizin, bacoside A, madecassoside and asiaticoside, in various in vitro cultures of medicinal plants have been published in recent years [12], however, much fewer data are available on the effect of jasmonate treatment on entire plants, and some have led to ambiguous conclusions, such as in the case of the trials of enhancement of steroidal saponin accumulation in *Dioscorea zingiberensis*, which revealed some physiological alterations relevant to antioxidant enzymes, stomatal traits and photosynthetic efficiency [26]. 

The aim of the present study was to evaluate the metabolic shifts between steroids and triterpenoids triggered by jasmonic acid in two experimental models, namely *C. officinalis* hairy root cultures and greenhouse cultivated plants (pot plants). Special attention was given to the comparison of the course of metabolic modifications in plants and hairy root cultures, the impact on biomass, as well as steroid biosynthesis modulations and changes in their conjugate forms (esters and glycosides) in the context of the possible role of these compounds in specific restructuring of membrane composition appearing in response to stress factors.

## 2. Results

### 2.1. Jasmonic Acid Influence on C. officinalis HairyRoot Culture

The elicitation of a *C. officinalis* hairy root culture was performed as described in Section 4.2.1. The 100 µmol/L concentration of exogenous jasmonic acid applied as elicitor was chosen as optimal regarding the results of the previous studies [11] to avoid a decay of the roots and to allow the prolonged duration of the experiment. The additional control treated with the adequate volume of 70% ethanol was set up to establish the influence of this solvent on the hairy root culture. The growth parameters and the contents of steroids and triterpenoids were determined at 7, 14, 21 and 28 days after treatment. Microscopic observations of the hairy roots were performed prior to the elicitation, and after 21 days of the experiment.

#### 2.1.1. Effect of Jasmonic Acid on Hairy Root Biomass

**Figure 1:**
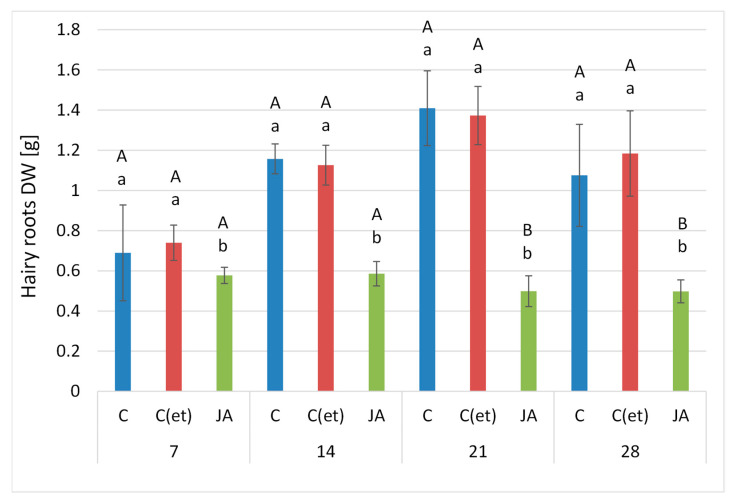
Time-dependent effect of jasmonic acid on hairy root biomass. C—control, C(et)—control samples supplemented with 70% ethanol, JA—jasmonic acid-elicited samples. Bars which do not share a common letter are significantly different. Capital letters indicate significant difference in time between plants from the same treatment, lowercase indicate difference between treatments within certain time point.

**Figure 2:**
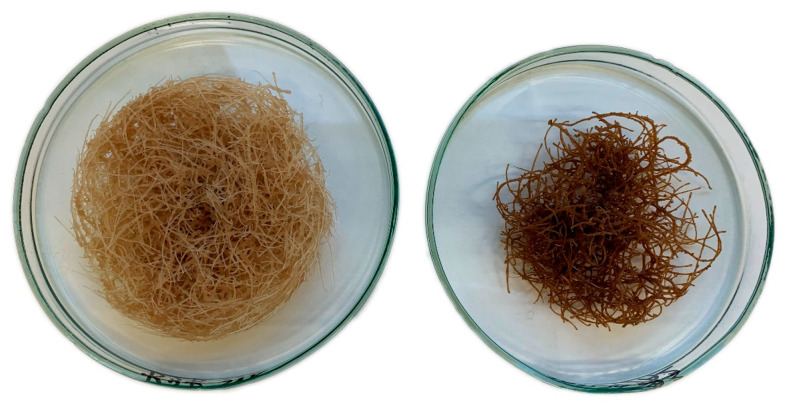
The morphological changes induced by jasmonic acid in *C. officinalis* hairy roots after 21 days of treatment. (**left**) control, (**right**) JA elicited sample.

The presence of jasmonic acid in the culture medium significantly altered the growth and appearance of *C. officinalis* hairy roots. The time-dependent effect of the applied elicitor on the hairy root biomass (expressed as dry weight, DW) is shown in Figure 1. The growth of elicited samples was visibly reduced, since their biomass remained almost unchanged throughout the experiment. After 7 days of elicitation, the difference in the D.W. between the jasmonic acid-treated samples and the control hairy roots was only slight (16% decrease), but this effect became stronger during the incubation period, when the difference between treated and control samples increased to 49%, 65% and 54% after 14, 21 and 28 days of elicitation, respectively. The reduced biomass growth of the elicited hairy roots was accompanied with alterations in their branching and the visible darkening of the tissues (Figure 2); elicited samples became dark brown already at 14 days after treatment.

From the beginning of the experiment, the control hairy roots were progressively growing, and doubled their biomass between 7 and 21 days of the experiment (Figure 1). However, at the end of the experiment (at 28 days) their biomass diminished by 23% and the visible process of the tissue darkening started, pointing to the beginning of the decay of the culture. The supplementation of the hairy root culture with adequate volume of 70% ethanol had no significant influence on the growth and appearance of the hairy root culture. Their biomass was comparable to the respective biomass of the control samples, and was even slightly higher after 7 and 28 days of the experiment (by 9% and 10%, respectively); however, these differences were not statistically significant.

#### 2.1.2. Microscopic Analysis of Hairy Root Tissues

**Figure 3:**
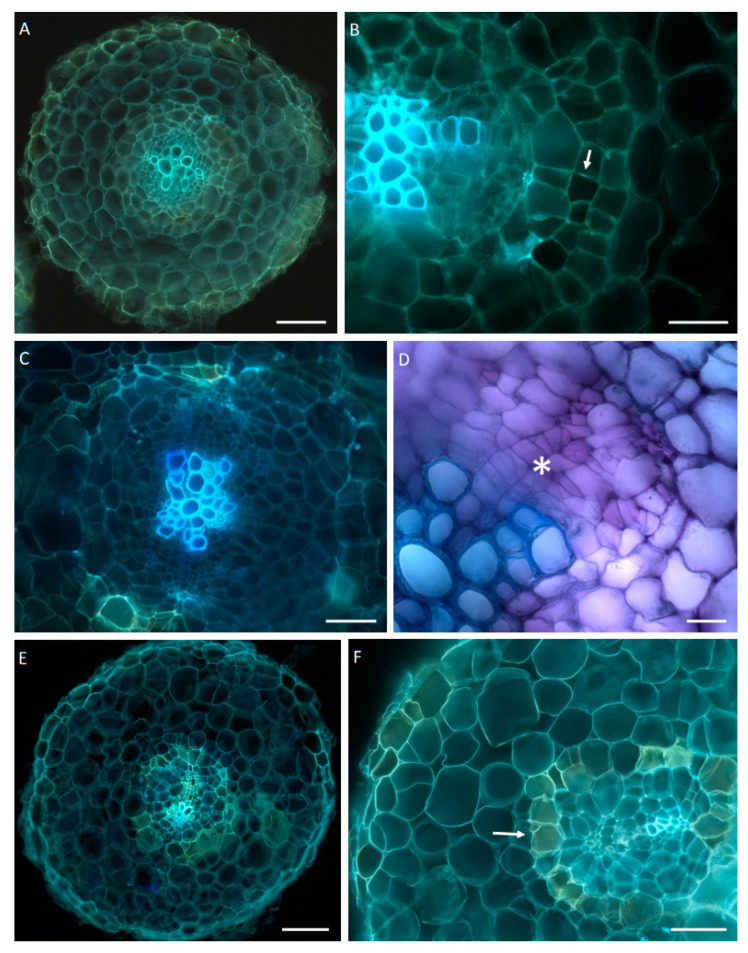
Anatomical structure of the roots—transverse sections. (**A**–**C**,**E**,**F**)—autofluorescence, UVB; (**D**)—bright field microscopy, staining with TBO. (**A**) The three-week-old root, primary structure. Note intact vascular cylinder and cortex. Scale bar—100 µm. (**B**) Detail of vascular cylinder and endodermis with Casparian bands (arrow). No cambial initials are present. Scale bar—50 µm. (**C**) The control root after 21 days of experiment with cambial initials and disrupted endodermis. Scale bar—50 µm. (**D**) Detail of cambial initials (asterisk) above strands of primary xylem. Scale bar—20 µm E. The jasmonic acid-treated root after 21 days of experiment. No visible signs of secondary growth. Scale bar—100 µm. F. Detail of vascular cylinder and intact endodermis with lignified cell walls (arrow). Scale bar—50 µm.

The initial samples of three-week-old roots prepared for the elicitation demonstrated typical primary structure (Figure 3A,B). In the control roots after 21 days of the experiment, the formation of secondary growth was evident (Figure 3C,D), whereas the jasmonic acid-treated roots still retained primary structure; however, on the contrary to the control, their endodermis was composed of cells having lignified cell walls (Figure 3E,F).

#### 2.1.3. Effect of Jasmonic Acid on the Content of Steroids

**Figure 4:**
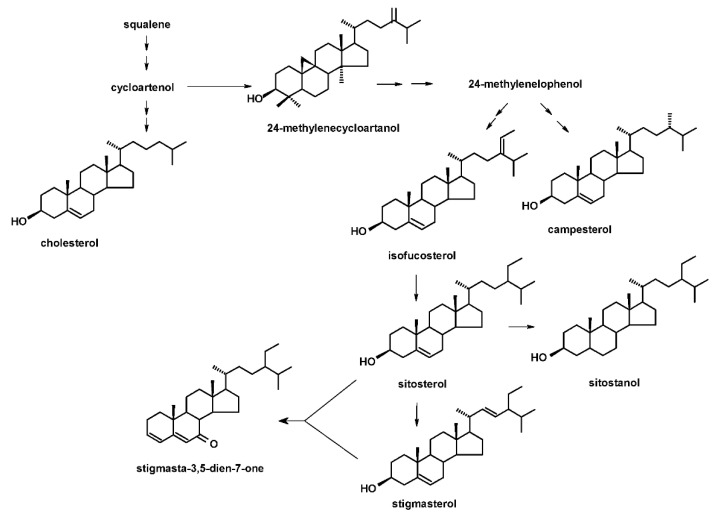
The steroids identified in the *C. officinalis* hairy root culture organized in the simplified putative biosynthetic pathway. Important intermediates occurring at pathway branching points are incorporated in the scheme only as names.

The obtained diethyl ether extracts of hairy roots were fractionated chromatographically as described in Section 4.4. Based on GC-MS analysis of the fraction of the free steroids, the compositions of these compounds were the same as described previously [27], i.e., the main sterols identified in this fraction were campesterol, cholesterol, isofucosterol, sitosterol, accompanied by its saturated form, sitostanol, and the predominating stigmasterol. Again, one steroid ketone, tremulone (stigmasta-3,5-dien-7-one), and one biosynthetic precursor, 24-methylenecycloartanol, were also identified. The GC parameters and respective MS spectra of all analyzed sterols and steroids are included in Table S1. The structures of the identified compounds, organized in the presumable biosynthetic pathway, are presented in Figure 4.

**Figure 5:**
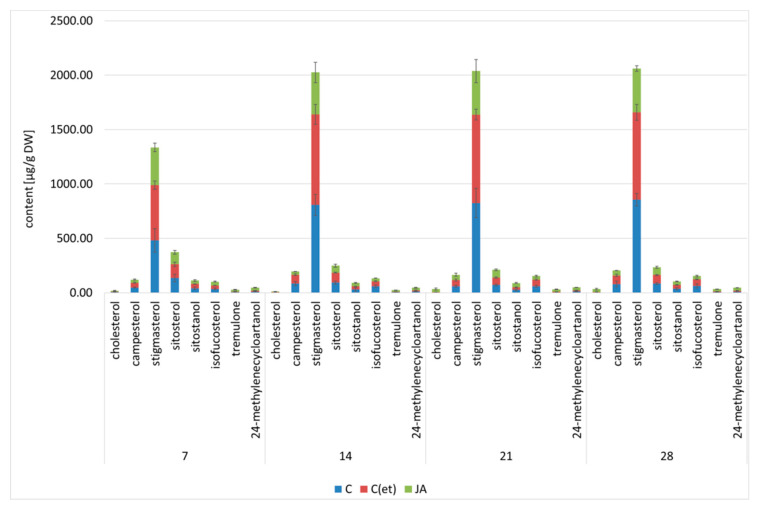
The effect of jasmonic acid on steroid content. C—control, C(et)—control samples supplemented with 70% ethanol, JA—jasmonic acid-elicited samples. See detailed data and statistical significance in Table S2.

Jasmonic acid elicitation resulted in a significant decrease in the total steroid content in hairy roots (Figure 5, Tables S2 and S3). The inhibition of steroid biosynthesis was evident throughout the experiment, starting with a 20% decrease in steroid content after 7 days of the treatment; reaching the extreme effect, i.e., almost two-fold decrease, after 14 days; and finishing with a 37% and 42% decrease after 21 and 28 days of the treatment, respectively. Supplementation with adequate volumes of 70% ethanol exerted practically no effect on the steroid content of hairy root cultures.

Although the sterol profile remained unchanged in elicited and control samples, the ratio between individual compounds, including the predominating stigmasterol and the following sitosterol, considerably differ. The content of stigmasterol decreased sharply in jasmonic acid-treated hairy roots, more than two-fold in samples after 14, 21 and 28 days of treatment, whereas the content of sitosterol was not as much affected (the maximal effect, i.e., the decrease by 32%, was noticed after 14 days of the treatment; in contrast, after 7 and 28 days of the experiment, the decrease was only 18% and 14%, respectively, and after 21 days of treatment there was no difference in sitosterol content between treated and untreated samples). According to these results, the characteristic stigmasterol-to-sitosterol ratio was visibly lower in the hairy roots elicited with jasmonic acid, e.g., after 21 days of treatment it equaled almost 12:1 in the control samples, whereas only 6:1 in the elicited samples.

Despite the remarkable decrease in total sterol content in elicited samples, elevated levels of some compounds, particularly cholesterol, tremulone and 24-methylenecyclo- artanol, were observed. The particularly significant effect was noticed after 21 days of treatment, when the content of cholesterol increased up to seven-fold in the elicited hairy roots, and the content of tremulone increased up to six-fold. The content of 24-methelenecycloartanol increased up to three-fold, particularly remarkably after 7 and 28 days of treatment.

The ester and glycoside fractions of diethyl ether and methanol extracts subjected to hydrolysis consisted of four sterols, i.e., campesterol, cholesterol, stigmasterol and sitosterol, as described in the previous report [24] (Section 4.5, Section 4.6 and Section 4.7, Figures S1 and S2, Tables S4–S6). In ester forms, the content of sterols was elevated gradually in elicited samples during the first 3 weeks of the experiment, mainly due to the significant increase in sitosterol content, that became the predominant compound (Figure S1). After 21 days of incubation with jasmonic acid, the content of sterols in the ester fraction was higher by 24% in the elicited samples as compared to the control. Finally, after 28 days of the experiment, the total sterol ester content in the jasmonic acid-treated hairy roots was found to be lower by 40% than that of the control, mainly due to the significant decrease of the content of stigmasterol, whereas the level of sitosterol was similar in the treated and untreated samples.

The content of sterol glycosides was much more affected and significantly higher in jasmonic acid-elicicted samples throughout the whole experiment (Figure S2). Already after 7 days of incubation, the total sterol content had increased more than two-fold in the elicited samples, and even more sharply after 14 days by almost four-fold. After 21 days, the total content of sterol glycosides was still higher in the treated samples (by 82%), but at the end of the experiment, after 28 days of incubation, this difference diminished to only 4%. In contrast to the sterol ester fraction, sitosterol was the most abundant among sterols occurring as glycoside conjugates only at the beginning of the experiment, after 7 days of the experiment, whereas with further incubation, stigmasterol was the predominating compound.

Again, as in the case of steroids occurring in the free form, supplementation of hairy root cultures with adequate volumes of 70% ethanol exerted practically no effect on the contents of the conjugate forms of sterols.

#### 2.1.4. Effect of Jasmonic Acid on the Content of Triterpenoids

**Figure 6:**
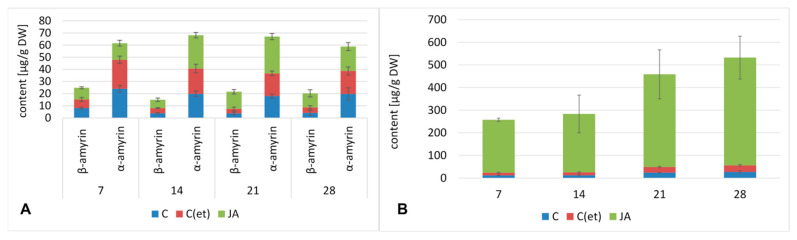
The effect of jasmonic acid on the content of neutral triterpenoids (amyrins) (**A**) and free oleanolic acid (**B**) in hairy roots tissue. C—control, C(et)—control samples supplemented with 70% ethanol, JA—jasmonic acid-elicited samples. See detailed data and statistical significance in Tables S7 and S9.

As previously reported, two triterpenoid alcohols were found in diethyl ether extracts from hairy roots, α-amyrin and β-amyrin, the precursors of ursane and oleanane type skeletons, respectively. The total content of both compounds decreased in the beginning of the experiment by 28% in jasmonic acid-treated roots compared to the control; however, with further incubation it significantly increased, by 48% and 35% after 14 and 28 days of incubation, respectively, and particularly remarkably—more than two-fold—after 21 days of the experiment (Figure 6A, Tables S7 and S8). The predominant compound was α-amyrin; its content decreased by 43% after the first 7 days of incubation, then increased by 48% and 68% after 14 and 21 days, respectively, and finally decreased practically to the level of the control samples. On the contrary, the level of β-amyrin, the precursor of oleanolic acid, was higher in the elicited samples from the beginning of the experiment; at first only slightly (by 18%) after 7 days of the experiment, but more significantly in the following weeks, i.e., by 79% after 14 days, and finally by almost five-fold and three-fold after 21 and 28 days of incubation, respectively.

The observed increase in β-amyrin biosynthesis in jasmonic acid-treated hairy roots was accompanied by the boost of the content of free oleanolic acid (Figure 6B, Tables S9 and S10). Indeed, the increase in the content of the free oleanolic acid content was remarkable, reaching a level approx. twenty-fold higher in elicited samples compared to the control throughout the experiment.

**Figure 7:**
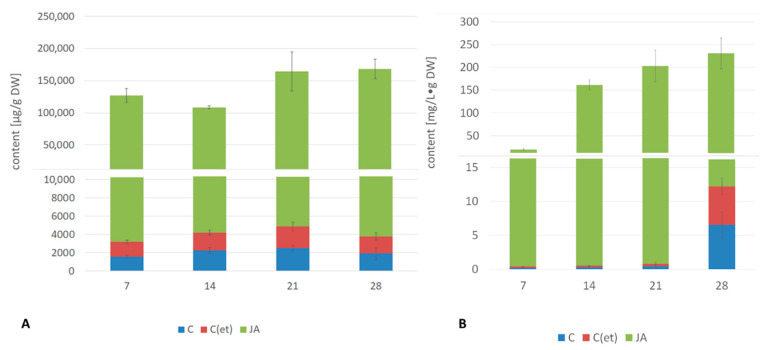
The effect of jasmonic acid on the content of saponins in the root tissue (**A**) and saponins released to the culture medium (**B**). C—control, C(et)—control samples supplemented with 70% ethanol, JA—jasmonic acid-elicited samples. See detailed data and statistical significance in Tables S11 and S13.

However, the most spectacular effect of jasmonic acid treatment was exerted on the biosynthesis of oleanolic acid saponins, both the compounds accumulated in the hairy root tissue (Figure 7A, Tables S11 and S12) as well as being released to the medium (Figure 7B, Tables S13 and S14). The increase in the level of saponin accumulation ranged during the experiment from 50-fold to more than 80-fold (the highest saponin content, increased by 86-fold, was noticed after 28 days of incubation. The boost of the saponin release to the medium was even more spectacular, starting from 91-fold after 7 days of incubation, reaching the maximum, i.e., 533-fold and 453-fold after 14 and 21 days of the experiment, respectively.

### 2.2. Jasmonic Acid Elicitation Effect on C. officinalis Plants

Three-week-old *C. officinalis* plants growing in the greenhouse were sprayed with 100 μM solution of jasmonic acid as described in Section 4.2.2. Control plants were sprayed with water (C) or water with adequate volume of 70% etanol (C(et)). Growth parameters and the contents of steroids and triterpenoids were determined separately in roots and aerial parts (shoots) after 7 and 14 days of cultivation.

#### 2.2.1. Jasmonic Acid Effect on Root and Shoot Growth Parameters

**Figure 8:**
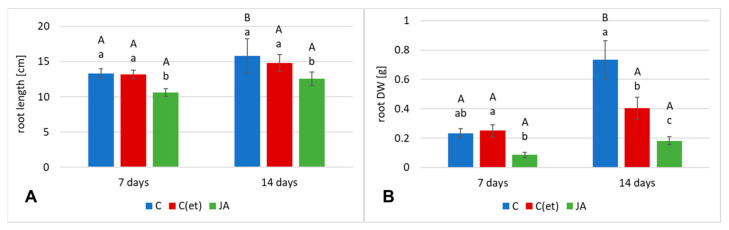
The length (**A**) and dry weight DW (**B**) of *C. officinalis* plant roots. C—control, C(et)—control samples treated with the adequate volume of 70% ethanol, JA—jasmonic acid-elicited samples. Bars which do not share a common letter are significantly different. Capital letters indicate significant difference in time between plants from the same treatment, lowercase indicate difference between treatments within certain time point.

Jasmonic acid elicitation had a remarkable impact on *C. officinalis* plant growth what was manifested by a decrease in length and biomass (DW) of roots and shoots. After 7 days of the experiment, the root lengths of the elicited samples were shorter by 20% than that of the control plants (Figure 8A), and DW decreased by 63% (Figure 8B). After 14 days, the root length was still 20% shorter, and the DW decreased by 75% in comparison to the control. The decrease in DW can be explained by the higher degree of hydration of the tissues of roots of the elicited plants. In the control samples C(et) (treated with water containing 70% ethanol) the influence on the growth parameters was not as strong as in the case of jasmonic acid treatment. After 7 days, the root length in C(et) samples was almost the same as in the control samples sprayed with water, and root DW slightly increased (by 7%, although this increase was not statistically significant) (Figure 8A,B). After 14 days, the root length in C(et) slightly decreased (by 6%) and the root DW decreased by 55%. This decrease in DW can be explained by observed changes in the morphology of the root system (thinner and less numerous lateral roots), probably also combined with the changes in water content in root tissues.

**Figure 9:**
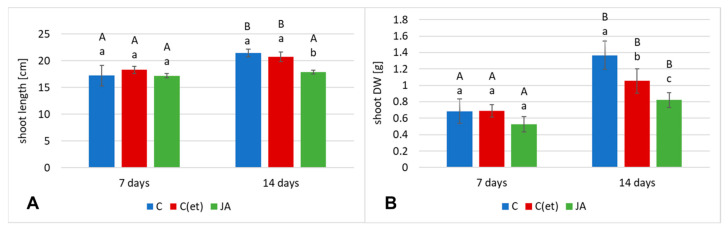
The length (**A**) and dry weight DW (**B**) of *C. officinalis* plant shoots. C—control, C(et)—control samples treated with the adequate volume of 70% ethanol, JA—jasmonic acid-elicited samples. Bars which do not share a common letter are significantly different. Capital letters indicate significant difference in time between plants from the same treatment, lowercase indicate difference between treatments within certain time point.

**Figure 10:**
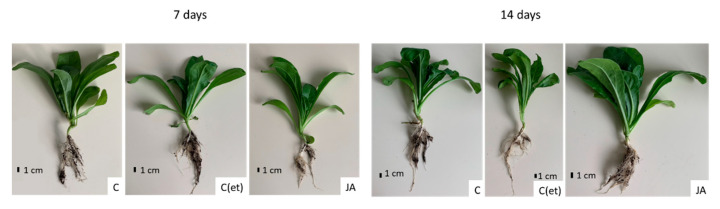
Control and JA—treated plants after 7 and 14 days of cultivation.

In *C. officinalis* shoots, the stronger influence of jasmonic acid elicitation was noticed on their biomass (DW) than their length. After 7 days of the experiment, the length of the control and elicited plants remained similar (Figure 9A), but the DW of the shoots of the jasmonic acid-elicited plants decreased by 23% (Figure 9B). After 14 days, the length of the shoots of the elicited and C(et) plants decreased by 17% and 3%, respectively, whereas their DW by 40% and 23%, respectively (Figure 9B). Photographs of *C. officinalis* plants after 7 and 14 days of cultivation are presented in Figure 10.

#### 2.2.2. Effect of Jasmonic Acid on the Content of Steroids in *C. officinalis* Roots

As previously reported [26], the steroid profile of *C. officinalis* roots consisted of cholesterol, campesterol, predominant stigmasterol, sitosterol and its fully hydrogenated derivative, sitostanol, three steroidal ketones (tremulone, sitostenone, stigmastane-3,6-dione), and one derivative of sterol precursor, cycloartenol acetate. In contrast to the steroid profile of the hairy roots, neither isofucosterol nor 24-methylenecycloartanol were detected.

**Figure 11:**
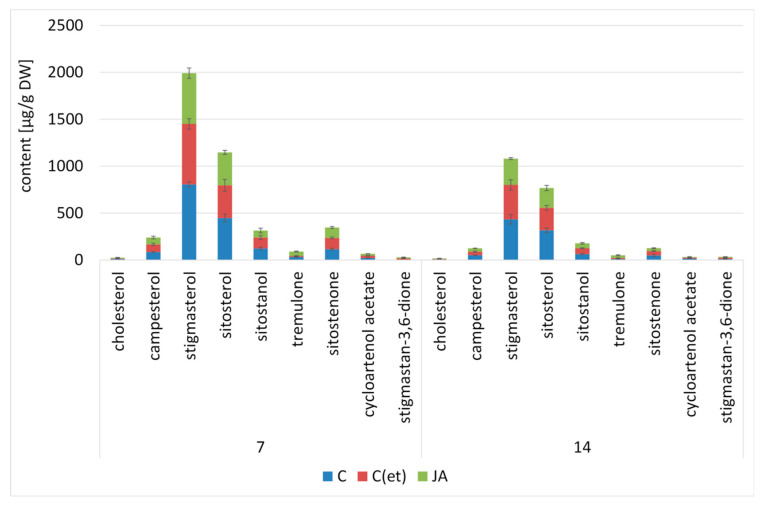
The content of free sterols in *C. officinalis* roots. C—control, C(et)—control samples treated with the adequate volume of 70% ethanol, JA—jasmonic acid-elicited samples. See detailed data and statistical significance in Table S15.

The influence of jasmonic acid treatment exerted on the biosynthesis and accumulation of steroids in *C. officinalis* plants was similar to the effect observed previously in hairy root cultures. After 7 and 14 days of the experiment, the total steroid content in the roots of jasmonic acid-elicited plants decreased by 17% and 30%, respectively, compared to the roots of the control plants sprayed only with water. The total steroid content in the C(et) samples also decreased by 17% both after 7 and 14 days of the experiment (Figure 11, Tables S15 and S16).

The changes in stigmasterol-to-sitosterol ratio induced by jasmonic acid treatment were not as remarkable as previously noticed in the hairy roots. After 7 days, the stigmasterol-to-sitosterol ratio equaled 1.8:1 in control samples, and 1.5:1 in the roots of jasmonic acid-treated plants. After 14 days of the experiment, this difference was even less noticeable, i.e., the stigmasterol-to-sitosterol ratio equaled 1.4:1 and 1.3:1 in control and elicited samples, respectively.

Similarly to the hairy roots, four sterols, i.e., cholesterol, campesterol, stigmasterol and sitosterol were found in conjugated (ester and glycoside) forms (Figure S3A,B, Tables S19 and S20). The total content of sterol esters did not change markedly after jasmonic acid treatment; it decreased slightly (by 8%) in the roots of the elicited plants after 14 days of the experiment. The proportion among sterols occurring in ester forms differed from the main profile of free sterols, namely, in both controls as well as in the elicited samples, the predominating compound cholesterol. Moreover, the content of all esterified sterols—except for cholesterol—decreased in the elicited roots, whereas cholesterol content increased by 40%. After 14 days of the experiment, cholesterol content was even more elevated (by 84%) in the elicited samples as compared to the control, whereas the content of other sterols decreased by 31% (Figure S3A).

The total content of sterol glycosides in the roots of jasmonic acid-treated plants was almost two-fold higher than in the control samples both after 7 and 14 days of experiment (Figure S3B). It was noteworthy that after 7 days of the experiment, the content of campesterol increased significantly (four-fold) in the jasmonic acid-treated samples, whereas sitosterol was the most abundant compound in both control samples. After 14 days of the experiment, sitosterol became the predominant compound in the roots of the elicited plants similarly to the control samples; however, the level of campesterol was still elevated by more than two-fold.

#### 2.2.3. Effect of Jasmonic Acid on the Content of Steroids in *C. officinalis* Shoots

**Figure 12:**
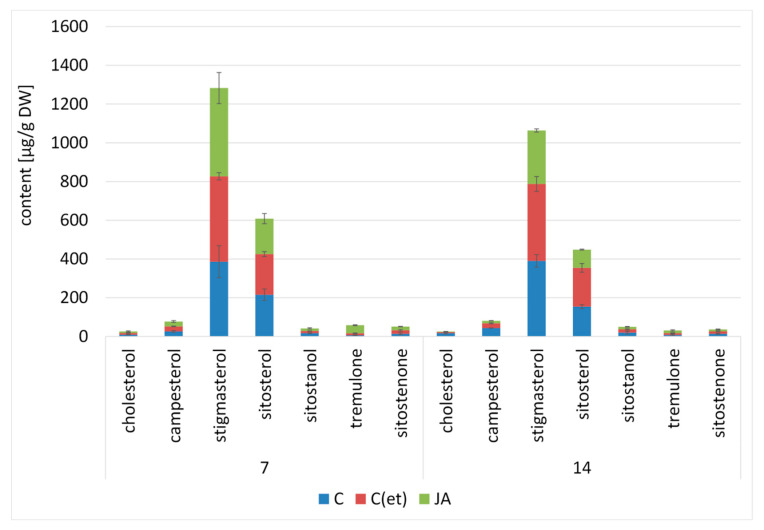
The content of free sterols in *C. officinalis* shoots. C—control, C(et)—control samples treated with the adequate volume of 70% ethanol, JA—jasmonic acid-elicited samples. Bars which do not share a common letter are significantly different. See detailed data and statistical significance in Table S21.

The profile of steroids present in free forms in *C. officinalis* shoots consisted of cholesterol, campesterol, stigmasterol, sitosterol accompanied by sitostanol, and two ketones, tremulone and sitostenone The total content of steroids increased slightly (by 11%) in shoots of jasmonic acid-treated plants after 7 days of the experiment (Figure 12, Tables S21 and S22).

A slight increase (by 7%) was also noted in the C(et) samples. However, after 14 days, the total steroid content in the elicited shoots decreased by 34% (similarly as in roots), while in the C(et) samples, it was comparable to the control. The dominant sterol was stigmasterol, followed by sitosterol, and the ratio of these compounds equaled 1.8:1 in the control samples, and increased to 2.5:1 in jasmonic acid-elicited shoots after 7 days of the experiment. After 14 days, the stigmasterol-to-sitosterol ratio in the shoots of the control plants increased to 2.5:1; however, in the elicited plants, it still remained higher than in the control (2.9:1).

The total content of sterols occurring in the ester form decreased after 7 and 14 days of the experiment by 25% and 20%, respectively, in the shoots of the jasmonic acid-treated plants as compared to the control (Figure S4A, Tables S25 and S26). Similarly to the roots, cholesterol was the predominating compound in the elicited shoots, and its content increased by three-fold as compared to the control samples.

The total content of sterol glycosides also decreased in the shoots after jasmonic acid elicitation, by 64% and 47% after 7 and 14 days, respectively (Figure S4B, Tables S25 and S26). However, the contents of esterified cholesterol and campesterol were elevated approx. two-fold in the jasmonic acid-treated shoots as compared to the control samples after 7 days of the experiment. Moreover, campesterol was the predominating compound in the elicited shoots, as observed previously in the case of the roots of the jasmonic acid-treated plants. After 14 days of the experiment, stigmasterol followed by sitosterol became the most abundant compounds in the elicited shoots, however, the content of cholesterol was still elevated twofold compared to the control samples.

#### 2.2.4. Effect of Jasmonic Acid on the Content of Triterpenoids in *C. officinalis* Roots and Shoots

**Figure 13:**
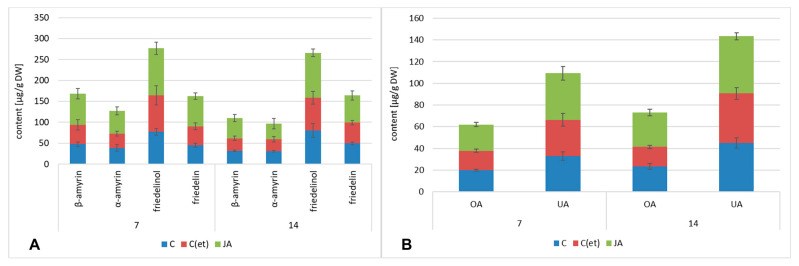
The content of neutral triterpenoids (**A**) and free acids (**B**) in *C. officinalis* roots. C—control, C(et)—control samples treated with the adequate volume of 70% ethanol, JA—jasmonic acid-elicited samples. See detailed data and statistical significance in Table S15.

The neutral triterpenoid profile of *C. officinalis* roots consisted of three triterpenoid alcohols, α-amyrin, β-amyrin and friedelinol (the most abundant alcohol in this fraction), and one ketone, friedelin, as described previously [24]. The total content of the neutral triterpenoids in the roots of the plants elicited with jasmonic acid increased by 51% and 34% after 7 and 14 days of the experiment, respectively (Figure 13A, Tables S15 and S17). The content of β-amyrin was almost two-fold elevated in the elicited samples as compared to the control, what could be correlated with the observed increased biosynthesis of oleanolic acid (Figure 13B, Tables S15 and S18). Apart from oleanolic acid, its isomer, ursolic acid, was also identified in *C. officinalis* roots (Figure 13B). The total content of free acids in the jasmonic acid-elicited samples increased by 27% and 24% after 7 and 14 days, respectively.

**Figure 14:**
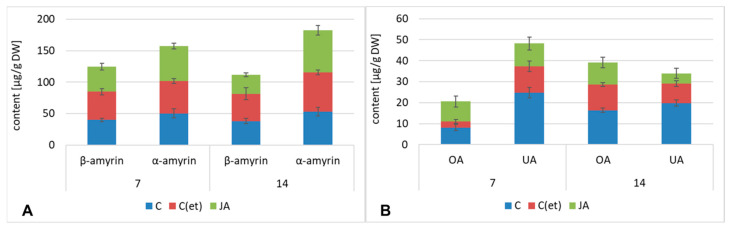
The content of neutral triterpenoids (**A**) and free acids (**B**) in *C. officinalis* shoots. C—control, C(et)—control samples treated with the adequate volume of 70% ethanol, JA—jasmonic acid-elicited samples. See detailed data and statistical significance in Table S21.

Only two amyrins were identified in the fraction of the neutral triterpenoids in *C. officinalis* shoots, i.e., α- and β-amyrin. Their total content increased only slightly, by 6% and 7% after 7 and 14 days, respectively, in jasmonic acid-elicited samples (Figure 14A, Tables S21 and S23); however, after 14 days, the decrease by 23% in β-amyrin content was noted. A slight increase in the content of amyrins was also observed in the case of the C(et) samples, indicating that ethanol treatment was not indifferent for *C. officinalis* plants.

Similarly to the roots, two triterpenoid acids occurring in a free form were identified in *C. officinalis* shoots: oleanolic and ursolic acid. The sum of both acids in the elicited shoots decreased by 38% and 58%, respectively, after 7 and 14 days of the experiment (Figure 14B, Tables S21 and S24). Their content was also lower in the C(et) samples, although not as remarkably as in jasmonic acid-treated shoots.

**Figure 15:**
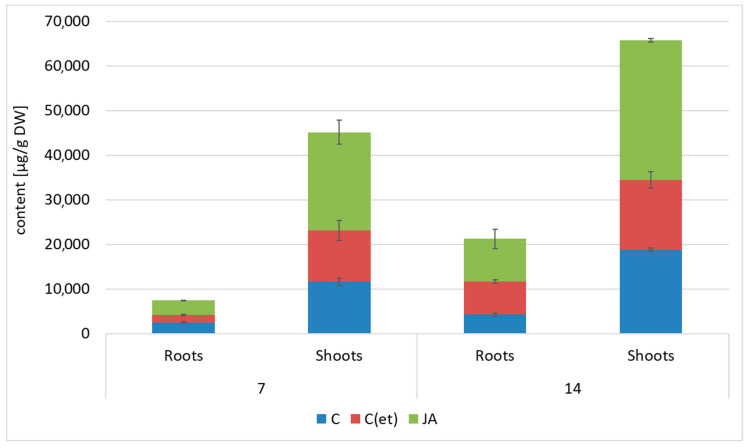
The content of saponins in roots and shoots of *C. officinalis* plants. C—control, C(et)—control samples treated with the adequate volume of 70% ethanol, JA—jasmonic acid-elicited samples. See detailed data and statistical significance in Table S27.

The exogenously applied jasmonic acid increased the biosynthesis of oleanolic acid saponins in both roots and shoots of *C. officinalis* plants (Figure 15, Tables S27 and S28).

Initially, after 7 days of the experiment, in the roots of the jasmonic acid-elicited plants, the saponin content increased only slightly, by 32%, but after 14 days this increase, at over twofold, was more significant. In the jasmonic acid-treated shoots, the saponin content increased almost two-fold compared to the control after 7 days of the experiments; however, after 14 days the difference between the saponin content in the elicited and control samples was less remarkable, at only 66%. After 14 days of the experiment, the saponin content increased by 75% in the roots of the C(et) samples as compared to the control plants treated with water, whereas it decreased by 17% in the C(et) shoots. This unexpected observation suggested that even small amount of ethanol could affect the saponin biosynthesis in both roots and shoots; however, this influence was different in both plant organs.

## 3. Discussion

The main goal of this study was to investigate the effect of an exogenously applied phytohormone, jasmonic acid, on steroid and triterpenoid metabolism in *C. officinalis* plants and hairy root cultures. Jasmonates are involved in a hormone-based defense mechanism against pathogens and herbivores, and act like “switchers” between general and specialized metabolisms, thereby redirecting metabolic flow toward defense compound biosynthesis and making them efficient elicitors in many in vitro and in vivo studies for valuable metabolite acquisition. However, various reports have indicated that plant defense response activation is often associated with growth inhibition [28,29,30,31]. Even though it remains unclear how growth rate is coupled with metabolic changes during jasmonate-induced defense mechanisms, this phenomenon has been reported for many plant species, such as sunflower (*Helianthus annuus*), tomato (*Solanum lycopersicum*), soybean (*Glycine max*), rice (*Oryza sativa*), and *Hypericum perforatum* [32,33,34]. In this *C. officinalis* study, growth inhibition upon jasmonic acid elicitation was observed in both hairy root cultures and pot plants. The previous initial study on hairy roots [11] also revealed a slight growth inhibition (approx. 20%) after seven days of incubation with jasmonic acid, which is confirmed by the current results (a hairy root D.W. decrease of 16%). However, the prolonged treatment caused a much stronger negative effect on biomass formation: more than twofold after 21 and 28 days of the experiment. The influence of jasmonic acid on hairy root growth and morphology was additionally confirmed by a microscopic study, which demonstrated symptoms of a lack of secondary growth and an increased cell wall lignification in jasmonic acid-treated samples. These observations indicate that the constant presence of jasmonic acid in the culture medium greatly affects the hairy root culture growth rate. This must therefore be considered in any biotechnological trial aiming to enhance specialized metabolite production by using exogenous jasmonic acid in plant in vitro cultures. However, if the yield of the desired specialized metabolites is the main aim of the culture, the inhibition of biomass formation can be ignored, and—particularly regarding the influence on triterpenoid saponin biosynthesis—jasmonic acid therefore remains one of the most efficient elicitors of specialized metabolite production.

A negative growth effect of exogenously applied jasmonic acid was also observed for the greenhouse-cultivated pot plants. Fourteen days after treatment, shoot and root length decreased by 17% and 20%, respectively, while biomass was more strongly affected; decreases of 40% and 75%, respectively, were noticed compared to the control plants. For a medicinal plant such as *C. officinalis*, cultivated for its bioactive compounds, growth inhibition can be considered less important than a significant enhancement in specialized metabolite content, which improves its quality as a herbal resource. For typical crop plants, this phenomenon can have negative consequences on general plant appearance and expected harvest yield; therefore, it is advised that the foliar application effects of any biostimulant containing jasmonates should first be investigated. However, different plant species react differently to jasmonate treatment, and its negative influence can be much weaker than for *C. officinalis*. Moreover, the effects of jasmonate treatment can progressively deteriorate during a prolonged cultivation time. For example, in one of the first field trial reports, tomato (*Lycopersicon esculentum*) plants treated with jasmonic acid developed fewer flowers than control plants, but neither plant appearance nor final yield were influenced, which was explained by an increased resistance to herbivore attack and the ability of tomato plants to modify the proportion of flowers that produce mature fruits [35]. Other reports revealed that exogenous jasmonic acid treatment decreased the photosynthetic rate of plants, caused a reduction in bud formation and accelerated climacteric fruit ripening by positively or negatively influencing ethylene production [36].

Sterols are integral membrane constituents and are required for proper cell division and plant growth. Therefore, it could be assumed that biomass formation suppression is connected to decreased sterol content. Indeed, the symptoms of sterol biosynthesis inhibition were noticed in the experiments, particularly in hairy root culture (which showed an extreme effect, i.e., an almost twofold decrease after 14 days of jasmonic acid treatment). A similar effect was observed in the pot plant roots (a decrease of up to 30% after 14 days after jasmonic acid treatment). However, the steroid biosynthesis reaction course, compared to jasmonic acid, was different in shoots, where a slight increase (11%) in total steroid content appeared seven days after foliar elicitor application, followed by a decrease (34%) after 14 days. Regarding a previous report on steroid metabolism modulation in *C. officinalis* hairy root cultures and plants exposed to cadmium stress [27], as well as metabolic changes in *Taxus* x *media* hairy roots triggered by jasmonic acid methyl ester [37], it might be concluded that sterol biosynthesis inhibition does not directly hamper plant growth induced by stress factors. On the contrary, in some cases, plant growth suppression can be inversely correlated with sterol content. Moreover, despite the observed decreasing trend in total steroid content in the hairy root cultures, a significant increase was noticed for one of the biosynthetic sterol precursors, 24-methylenecycloartanol (up to three-fold), which was paralleled by increased cholesterol biosynthesis (up to seven-fold). The stigmasterol to sitosterol ratio was modified in both hairy root cultures and pot plants elicited by jasmonic acid. All this suggests that the steroid biosynthetic pathway was specifically modified and not simply hampered due to jasmonic acid treatment. It can be assumed that the primary plant stress response mechanism involving sterol metabolism changes seems to be connected to a restructured membrane composition, and therefore to membrane property regulation, rather than a simple increase or decrease in sterol biosynthesis. The plant growth suppression that is often observed after jasmonate treatment can be explained as a common result of various general metabolic shifts, which includes not only sterols, but also other compounds as sugars and starch [38].

The jasmonic acid treatment significantly influenced the sterol conjugate proportions; however, this differed between the investigated experimental models. An increase in the investigated forms, esters, and glycosides was noticed in hairy root cultures. The accumulation of sterol glycosides increased in the pot plant roots, whereas the content of the ester forms remained practically unaltered, except for cholesterol content, which increased. In contrast, the sterol ester and sterol glycoside content decreased in pot plant shoots. Sterol esters and sterol glycosides are often considered to be a storage pool of sterols that serve plants during stressful conditions and acclimation processes; however, their precise role remains unknown and seems to vary according to plant species [39,40]. An increase in sterol glycoside content was also reported in a study on methyl jasmonate treated *Taxus* x *media* hairy roots [37].

Jasmonic acid elicitation strongly increased oleanolic acid biosynthesis in hairy root cultures, both in free form as well as oleanolic acid saponins that accumulated and were released into the culture medium. The 86-fold increase of saponin accumulation in hairy root tissue, combined with the 533-fold boost of the saponin release to the medium, are certainly among the most spectacular effects reported for the enhancement of triterpenoid saponin production in in-vitro cultures. An increase in free triterpenoid acids and oleanolic acid saponins occurred also in pot plant roots after jasmonic acid elicitation, however, two-fold stimulation of saponin biosynthesis was less remarkable than the phenomenon observed in the hairy root culture. This again hints at a correlation between the response of hairy roots cultures and native plant roots. For shoots, the free acid content decreased after jasmonic acid treatment, whereas the oleanolic acid saponin content increased sharply, which suggests that the triterpenoid glycosylation process is significantly stimulated during jasmonic acid elicitation. Summarizing elicitation responses of in vitro cultures and pot plants, four main conclusions can be drawn: (i) various organs in the same plant can react differently to jasmonic acid elicitation; (ii) hairy root cultures may be used as a useful in vitro model to track metabolic changes, but this should be carried out with caution; (iii) glycosylation (of both, triterpenoids and sterols) seems to be important strategy in jasmonic acid elicitation; and (iv) jasmonic acid elicitation notably decreases steroid content and increases specialized metabolite biosynthesis. It is noteworthy that only the above-ground plant parts were directly exposed to jasmonic acid treatment. The stress signal was therefore quickly transferred to the roots, where it induced a strong metabolic response.

Another aim of the present study was to evaluate how long the elicitation effect would last, and whether the treated plants would overcome stressful conditions, at least at the metabolomic level; in other words, whether or not the metabolic shift towards enhanced specialized metabolite biosynthesis would revert back to its basic course. Here, it was more convenient to compare in vitro cultures and pot plants separately. In hairy roots, the results indicated that trends continued and that differences between treated samples and controls increased with experimental duration, as with free oleanolic acid content in hairy root tissues, or saponins that accumulated in, and were released into, the culture medium. It is worth remembering that, in the case of in vitro cultures, jasmonic acid was added once and remained in the culture medium during the entire experimental duration. Single-spray treatment of pot plants seems to contrast with this, but it mimics numerous naturally occurring phenomena. A single treatment of pot plants with jasmonic acid was enough to cause sterol biosynthetic inhibition and to parallel specialized metabolite enhancement; however, this enhancement was not as remarkable and instantaneous as in hairy roots. It also seems that a 14-day period is not long enough for plants to overcome stressful conditions, and that jasmonic acid elicitation has a long-lasting effect. Many reports document the use of jasmonates as priming agents to induce long-lasting pest and plant disease resistance. These oxylipins are involved in a complex regulatory hub with other plant hormones such as ethylene, salicylic acid, or abscisic acid [41,42,43]. Complex interactions with other phytohormones during stress responses and metabolic reprogramming could explain why jasmonic acid elicitation is challenging for plants, and why it has such long-lasting effects.

In the current study, additional control samples treated with an adequate volume of ethanol were applied to evaluate the influence of this solvent on *C. officinalis* growth and metabolism. No significant effect was observed for hairy roots, but in contrast, a much larger effect was observed for pot plants. Most reports on the use of ethanol in jasmonic acid experiments claim that ethanol has no significant effect on the treated plants, but the current findings suggest that ethanol treatment (even in low concentrations) may indeed affect the plants. The data concerning the effects of ethanol foliar treatment on plants are scarce; however, published results indicate that this solvent may have a negative impact on some plant species. In a study where the foliar treatment of 20% and 40% ethanol solution was applied to lettuce (*Lactuca sativa*), plant length decreased, as well as the size of the lettuce heads [44]. A 10% ethanol foliar treatment on Dragonhead (*Dracocephalum moldavica* L., Lamiaceae) also decreased its height, biomass, and essential oil yield [45]. On the other hand, studies conducted on sweet basil (*Ocimum basilicum* c.v. Keshkeni luvelou) indicated that ethanol has biostimulating properties, since it increased biomass and yield [46]. The results obtained in the current study suggest that *C. officinalis* may belong to the group of plants that are sensitive to ethanol foliar application, even at low concentrations, although no traces of mechanical damage were observed after the treatment. The observed metabolic modifications can be explained by *C. officinalis* leaves that are covered with delicate glandular trichomes containing a mixture of essential oils which are released after mechanical damage or herbivore attack. Some of these released volatile compounds can induce a priming strategy within the plant, thereby influencing its metabolic pathways.

## 4. Materials and Methods

### 4.1. Plant Material

#### 4.1.1. Hairy Root Cultures

*C. officinalis* hairy roots line CC16 (derived from cotyledon explant) was obtained according to a previously described procedure [10]. The roots were cultivated in a ½ Murashige–Skoog liquid medium, at 23–25 °C, in darkness on a rotary shaker at 120 rpm. Subcultures were performed every 3–4 weeks by transferring the 1–2 cm pieces of the young, branched root to 100 mL of a fresh medium.

#### 4.1.2. Pot Cultures

*C. officinalis* seeds (PNOS, Ożarów Mazowiecki, Poland) were sown into rectangular plastic pots (dimensions: 80 × 18 × 14 cm) filled with universal flower soil “Athena” (the details of physical and chemical characterization are in Table S29). Germination of seed started within 3 days of sowing. The plants were cultivated for 3 weeks in the greenhouse under controlled conditions (16/8 h day/night photoperiod, 52 ± 2% humidity, temperature 20 °C with a light intensity of 120 ± 10 µmol/m^2^ s).

### 4.2. Elicitation with Jasmonic Acid

#### 4.2.1. Elicitation of Hairy Roots

Freshly sub-cultured roots were incubated for 21 days to obtain at least 1.5 g in fresh weight. Afterwards, they were weighed and transferred to 100 mL of fresh medium five days prior to elicitation. A stock solution of JA (Sigma J2500) was prepared by dissolving 250 mg in 70% (*v*/*v*) ethanol. Further dilutions were made in distilled water. The final solution was sterilized using a 0.22 μm syringe filter (Millipore, Bionovo, Legnica, Poland) and added to the culture medium in a concentration of 100 μM. Control cultures were treated with adequate volume of 70% ethanol. The hairy roots were cultured for 7, 14, 21 and 28 days.

#### 4.2.2. Elicitation of Pot-Cultures

Three-week-old marigold seedlings were sprayed with 100 μM jasmonic acid solution until run-off (ca. 13 mL per plant). Control plants were sprayed with distilled water (C) and distilled water with adequate to jasmonic acid volume of 70% etanol (C(et)). The plants were cultivated either for 1 week or for 2 weeks in the greenhouse under the conditions described above.

### 4.3. Extraction and Fractionation

#### 4.3.1. Extraction of the Hairy Roots and the Culture Medium

After 7, 14, 21 and 28 days of incubation with jasmonic acid, the culture media were filtered from the hairy roots. The harvested hairy roots were air-dried at room temperature before extraction, whereas the culture media were directly extracted 3 times with 40 mL portions of n-butanol to extract oleanolic acid saponins released to the medium. Dried hairy roots were powdered and extracted using a Soxhlet apparatus for 8 h with diethyl ether to obtain fractions of free sterols, conjugated sterols (esters and low polar glycosides), free triterpenoid acids and alcohols, and then 8 h with methanol to extract more polar sterol glycosides and oleanolic acid saponins. The obtained extracts were evaporated to dryness under reduced pressure on a rotary evaporator.

#### 4.3.2. Extraction of the Plants

After 7 and 14 days of growing after jasmonic acid treatment, plants were gently collected, then weighed, and lengths of the root and the shoots (aerial) parts were measured. Roots and aerial parts were left to dry in the dark and airy place, then weighed again, grinded in mortar to fine powder and extracted in Soxhlet apparatus for 8 h with diethyl ether and then 8 h with methanol. The obtained extracts were evaporated to dryness under reduced pressure on a rotary evaporator.

### 4.4. Fractionation of Diethyl Ether Extracts

Evaporated diethyl ether extracts obtained from the hairy roots, as well as roots and aerial parts of native plants were fractionated by adsorption preparative TLC on 20 cm × 20 cm glass plates coated manually with silica gel 60H (Merck, Darmstadt, Germany). The solvent system chloroform: methanol 97:3 (*v*/*v*) was applied for developing. Four fractions were obtained as described by Sykłowska-Baranek et al. [37]: (i) esters; (ii) free steroids and neutral triterpenoids; (iii) free triterpenoid acids; and (iv) glycosides [46]. Fraction (ii) containing free steroids and neutral triterpenoids (alcohols) was directly analyzed using a GC–MS, gas chromatography–mass spectrometer (Agilent Technologies 7890A); the triterpenoid acid fraction (iii) was methylated with diazomethane prior to GC–MS analysis as described previously [47]; the ester fraction (i) was subjected to alkaline hydrolysis to release the steroid core from ester forms; and the glycoside fraction (iv) to acidic hydrolysis to release the aglycones.

### 4.5. Alkaline Hydrolysis

The ester fractions were subjected to alkaline hydrolysis with 10% NaOH in 80% methanol at 80 °C for 3 h. Subsequently, 5 volumes of water were added to each hydrolysate, the pH was neutralized with 5% acetic acid, and the obtained mixtures were extracted with diethyl ether (3 × 20 mL) in a separation funnel. The extracts were evaporated and fractionated by preparative TLC (as described in Section 4.4) to obtain the fraction of sterols released from their ester forms.

### 4.6. Acidic Hydrolysis

The glycoside fractions obtained from the diethyl ether extracts, evaporated methanol extracts and n-butanol extracts from the culture medium were hydrolyzed by 11% HCl in 80% methanol during 2 h on a heating mantle under reflux [37,47]. Subsequently, the hydrolysates were diluted with distilled water, methanol was evaporated in a rotary evaporator, and the obtained aqueous remnants were extracted 3 times with 40 mL portions of diethyl ether in a separation funnel. The obtained extracts were washed with distilled water 3 times and evaporated to dryness.

### 4.7. Fractionation of Acidic Hydrolysates

The dried extracts obtained from acidic hydrolysates were divided by preparative TLC on 20 cm × 20 cm glass plates, manually coated with silica gel 60H (Merck, Darmstadt, Germany). The solvent system chloroform: methanol 95:5 (*v*/*v*) was used for developing the plates. From the hydrolyzed methanol extracts, two fractions were obtained: sterols and oleanolic acid. From the hydrolyzed n-butanol extracts, only one fraction was obtained: oleanolic acid. Purified oleanolic acid was methylated with diazomethane.

### 4.8. Derivatization of Triterpenoid Acids

Nitrosomethylurea (2.06 g) was added to a mixture of 20 mL of diethyl ether and 6 mL of 50% aqueous KOH, and the organic layer was then separated from the aqueous layer. Samples containing triterpenoid acids were dissolved in 2 mL of the obtained solution of diazomethane in diethyl ether, and held at 2 °C for 24 h.

### 4.9. Identification and Quantification of Triterpenoids by GC–MS/FID

An Agilent Technologies 7890 A gas chromatograph equipped with a 5975C mass spectrometric detector was used for qualitative and quantitative analyses. Samples dissolved in diethyl ether:methanol (5:1, *v*/*v*) were applied (in a volume of 1–4 μL) using 1:10 split injection. The column used was a 30 m × 0.25 mm i.d., 0.25 μm, HP-5MS UI (Agilent Technologies, Santa Clara, CA, USA). Helium was used as the carrier gas at a flow rate of 1 mL/min. The separation was made either under isothermal conditions at 280 °C or at the temperature programmed: an initial temperature of 160 °C held for 2 min, then increased to 280 °C at 5 °C/1 min and the final temperature of 280 °C held for further 44 min. The other employed parameters were as follows: inlet and FID (flame ionization detector) temperature 290 °C; MS transfer line temperature 275 °C; quadrupole temperature 150 °C; ion source temperature 230 °C; EI 70 eV; m/z range 33–500; FID gas (H2) flow 30 mL min^−1^ (hydrogen generator); and air flow 400 mL min^−1^. Individual compounds were identified by comparing their mass spectra with library data from Wiley 9th ED. and NIST 2008 Lib. SW Version 2010 or previously reported data and by comparison of their retention times and corresponding mass spectra with those of authentic standards, when available. Quantitation was performed using an external standard method based on calibration curves determined for the compounds belonging to representative triterpenoid classes: α-amyrin for triterpene alcohols, oleanolic acid methyl ester for triterpene acid methyl esters, and sitosterol for steroids.

### 4.10. Microscopic Photos of Hairy Roots

For microscopic analysis the material was fixed in 70% ethanol. Preliminary observations of the morphology of roots (thickness, branching, presence of root hairs) were examined using a Nikon SMZ1000 stereoscopic microscope. Afterwards, the roots were hand sectioned and subjected to anatomical investigations by bright-field light microscopy (LM) and fluorescence microscopy using a Nikon Eclipse Ni-U microscope equipped with a Prior 200W lamp (Prior Scientific Instruments Ltd., Cambridge, UK) and UV-2B cube filter 355/50 (330–380 nm excitation filter; a 400 nm (LP) dichroic mirror and a 435 nm (LP) barrier filter) to detect autofluorescence of cell walls. Additionally, hand-cut sections were stained for general histology using 0.05% Toluidine blue O [48]. Micrometry and photomicrography were carried out using a DS -Fi1 digital camera (Nikon, Japan) and NIS-Elements BR software.

### 4.11. Statistical Analysis of Data

All data are presented as the means ± standard deviation of three independent samples analyzed in triplicate. To identify significant differences between the control and elicitor-treated samples, a two-way analysis of variance (ANOVA) and Tukey’s multiple comparisons test were applied (*p* ≤ 0.05). Statistical modeling was performed using the Statistica™ software (Version 13.3, © 1984–2017 TIBCO Software Inc., Palo Alto, CA, USA).

## 5. Conclusions

Jasmonic acid is considered to be an effective elicitor in biotechnology (including various plant in vitro culture types) and is also applied as an inductor of natural plant resistance in modern sustainable agriculture. Therefore, the present investigation compared the course of metabolic modifications (involving steroids and triterpenoids) triggered by jasmonic acid in two experimental models, namely *C. officinalis* hairy root cultures and greenhouse-cultivated plants (pot plants). The effect exerted by jasmonic acid elicitation on hairy root cultures was particularly spectacular; the saponin production was enhanced up to 86-fold in root tissue, whereas saponin release to the medium increased by 533-fold. However, it is noteworthy that such phenomenon could occur only upon the constant influence of the elicitor, present in the medium throughout the experiment, and in the type of in vitro culture in which the release of the compounds to the medium is facilitated. The pot plants were treated with a single foliar application of jasmonic acid; therefore, the exerted effect, i.e., two-fold enhancement of saponin biosynthesis was less remarkable than in the case of hairy root cultures. Moreover, the obtained results revealed that various organs in the same plant, such as shoots and roots, can react differently to jasmonic acid elicitation. In both models, the increase in triterpenoid biosynthesis was parallel to the decrease in biomass formation, and modifications of the sterol content, involving stigmasterol-to-sitosterol ratio and the proportions between ester and glycoside conjugates. These observations suggested that the steroid biosynthetic pathway was specifically modified due to jasmonic acid treatment; and the changes in sterol metabolism seems to be connected to a restructured membrane composition, and therefore to the regulation of membrane properties, rather than a simple increase or decrease in sterol biosynthesis.

## Data Availability

The data presented in this study are available in the article and Supplementary Materials.

## Supplementary Materials

The following supporting information can be downloaded at: https://www.mdpi.com/article/10.3390/ijms232012173/s1.
