# Supplementary material for: The Influence of Exogenous Jasmonic Acid on the Biosynthesis of Steroids and Triterpenoids in Calendula officinalis Plants and Hairy Root Culture
Source: Int J Mol Sci. 2022 Oct 12;23(20):12173. doi: 10.3390/ijms232012173 (PMC9603384; doi:10.3390/ijms232012173)
Supplement: Supplementary file 1 [file ijms-23-12173-s001.zip › ijms-1928259-supplementary.pdf]

The Influence of Exogenous Jasmonic Acid on the Biosynthesis of Steroids and Triterpenoids in *Calendula officinalis* Plants and Hairy Root Culture

Table S1.GC-MS data (retention times and characteristic ions of mass spectra) of identified steroids and triterpenoids.

Compound	Formula	Molecular weight	Retention time[min]	Mass spectrum *m*/*z*(relative intensity)
cholesterol	$C_{27}H_{46}O$	386.6	31.06	386 (26), 107 (50), 105 (48), 91 (57), 81 (54), 79 (46), 69 (47), 57 (87), 55 (73), 43 (100), 41 (55)
campesterol	$C_{28}H_{48}O$	400.6	33.59	400 (30), 107 (51), 105 (55), 95 (49), 83 (45), 81 (64), 71 (62), 57 (77), 55 (77), 43 (100), 41 (52)
stigmasterol	$C_{29}H_{48}O$	412.6	34.52	412 (36), 145 (64), 107 (52), 95 (100), 83 (66), 81 (90), 78 (60), 69 (67), 67 (85), 55 (69)
sitosterol	$C_{29}H_{50}O$	414.7	36.15	414 (29), 145 (54), 107 (59), 105 (60), 95 (54), 91 (49), 81 (57), 57 (68), 55 (70), 43 (100)
sitostanol	$C_{29}H_{52}O$	416.7	36.40	416 (31), 215 (82), 109 (58), 107 (83), 95 (81), 93 (64), 81 (84), 69 (60), 57 (64), 55 (81), 43 (100)
isofucosterol	$C_{29}H_{48}O$	412.3	36.78	412 (5), 314 (100), 105 (47), 95 (50), 91 (42), 83 (40), 81 (51), 69 (61), 55 (96), 43 (49)
β-amyrin	$C_{30}H_{50}O$	426.7	37.13	426 (27), 219 (18), 218 (100), 203 (49), 189 (17), 135 (11), 109 (13), 105 (12), 95 (15), 81 (18), 69 (14)
α-amyrin	$C_{30}H_{50}O$	426.7	38.62	426 (4), 219 (18), 218 (100), 203 (20), 189 (19), 135 (17), 133 (15), 122 (16), 119 (15), 95 (16)
tremulone	$C_{29}H_{46}O$	410.7	39.20	410 (32), 187 (27), 174 (100), 161 (37), 159 (26), 91 (28), 57 (28), 55 (37), 43 (44), 41 (28)
sitostenone	$C_{29}H_{48}O$	412.7	40.72	412 (37), 229 (34), 218 (31), 124 (100), 109 (31), 95 (41), 81 (27), 69 (32), 55 (37), 43 (44)
cycloartenol acetate	$C_{32}H_{52}O_2$	469.8	41.9	468 (24), 121 (20), 109 (32), 107 (29), 95 (41), 93 (24), 81 (27), 69 (30), 55 (23), 43 (100)
friedelinol	$C_{30}H_{52}O$	428.7	42.7	428 (3), 125 (45), 123 (49), 121 (48), 109 (75), 107 (47), 96 (68), 95 (100), 81 (66), 69 (82)
friedelin	$C_{30}H_{50}O$	426.7	43.7	426 (6), 125 (65), 123 (78), 109 (82), 107 (46), 96 (62), 95 (94), 81 (77), 69 (100), 67 (56)
oleanolic acid methyl ester	$C_{31}H_{50}O_3$	470.1	46.37	470 (1), 262 (48), 207 (13), 204 (16), 203 (100), 202 (21), 189 (22), 133 (17), 119 (13), 105 (14
stigmastane-3,6-dione	$C_{29}H_{48}O_2$	428.6	48.03	428 (25), 135 (61), 107 (74), 98 (63), 95 (67), 79 (62), 69 (86), 57 (67), 55 (100), 43 (77), 41 (71)
ursolic acid methyl ester	$C_{31}H_{50}O_3$	470.1	48.96	470 (1), 263 (20), 262 (100), 207 (32), 203 (93), 189 (29), 133 (76), 119 (34), 105 (21), 95 (18)

Table S2. Effect of jasmonic acid elicitation on steroid content in hairy roots tissue. Data which do not share a common letter are significantly different. Capital letters indicate significant difference in time between plants from the same treatment, lowercase indicate difference between treatments within certain time point.

Compound	Content [µg/g DW± SD]											
	days											
	7			14			21			28		
	C	C(et)	JA	C	C(et)	JA	C	C(et)	JA	C	C(et)	JA
cholesterol	4.53±2.14 A, a	4.72±1.53 A, a	10.44±2.13 A, a	1.91±1.19 A, a	2.22±1.01 A, a	7.37±1.03 A, a	3.78±1.50 A, a	2.82±0.37 A, a	27.05±9.70 B, b	3.59±2.45 A, a	3.26±0.54 A, a	25.83±8.88 B, b
campesterol	47.83±4.87 A, a	44.41±4.06 A, a	27.96±4.70 A, a	84.56±17.73 B, a	80.88±9.04 B, a	30.17±1.02 A, b	59.06±9.50 AB, a	56.71±9.66 AB, a	49.59±14.20 A, a	79.24±26.40 AB, a	79.69±10.09 AB, a	44.96±2.62 A, a
stigmasterol	481.27±107.14 A, a	508.85±37.72 A, a	345.69±38.64 A, a	806.84±97.76 B, a	832.36±92.85 B, a	386.41±93.93 A, b	825.16±135.97 B, a	811.86±49.61 B, a	401.53±106.51 A, b	854.85±57.15 B, a	803.49±73.29 B, a	402.95±25.78 A, b
sitosterol	135.20±35.68 A, a	126.87±20.27 A, a	110.00±16.07 A, a	93.33±13.58 AB, a	90.97±4.05 AB, a	63.89±15.75 B, a	70.59±6.70 B, a	69.06±6.25 B, a	71.33±8.34 AB, a	83.68±7.00 B, a	78.85±3.70 B, a	71.47±10.29 AB, a
sitostanol	41.34±10.34 A, a	38.97±2.09 A, a	31.86±7.14 A, a	31.69±15.79 A, a	27.82±1.34 A, a	30.54±5.14 A, a	26.34±8.04 A, a	24.64±3.07 A, a	38.15±5.34 A, a	38.03±8.94 A, a	33.52±3.25 A, a	31.81±3.08 A, a
izofucosterol	34.78±7.24 A, a	32.48±0.91 A, a	34.46±4.92 A, a	58.88±12.71 AB, a	46.52±4.19 AB, ab	28.10±2.59 A, b	60.56±9.45 B, a	58.28±6.76 B, ab	34.28±8.89 A, b	61.84±18.55 B, a	61.14±4.22 B, a	30.46±6.87 A, b
tremulone	4.77±1.73 A, a	4.77±0.95 A, a	18.74±4.02 AB, b	4.43±0.71 A, a	3.53±1.14 A, a	15.37±1.62 B, b	3.74±1.19 A, a	3.59±0.84 A, a	23.52±2.10 A, b	5.86±2.97 A, a	4.89±1.03 A, a	22.48±0.81 A, b
24-methylene cycloartenol	9.86±2.48 A, a	9.00±0.98 A, a	28.21±4.05 A, b	11.53±2.59 A, a	9.05±4.00 A, a	24.88±4.99 A, b	12.25±2.62 A, a	9.66±2.53 A, a	26.49±2.15 A, b	9.75±6.50 A, a	9.82±0.37 A, a	27.18±0.79 A, b
**Total**	**759.58**	**770.07**	**607.38**	**1093.17**	**1093.35**	**586.73**	**1061.49**	**1036.62**	**671.94**	**1136.84**	**1074.65**	**657.13**

Table S3. Analysis of the interaction of treatment and time on steroid content in hairy roots tissue performed by two-way ANOVA.

	p value							
	cholesterol	campesterol	stigmasterol	sitosterol	sitostanol	isofucosterol	tremulone	24-methylenecycloartanol
treatment	<0.001	<0.001	<0.001	<0.05	n.s.	<0.001	<0.001	<0.001
time	0.002	<0.001	<0.001	<0.001	n.s.	0.001	0.007	n.s.
treatment x time	<0.001	n.s.	<0.05	n.s.	n.s	0.024	0.007	n.s.

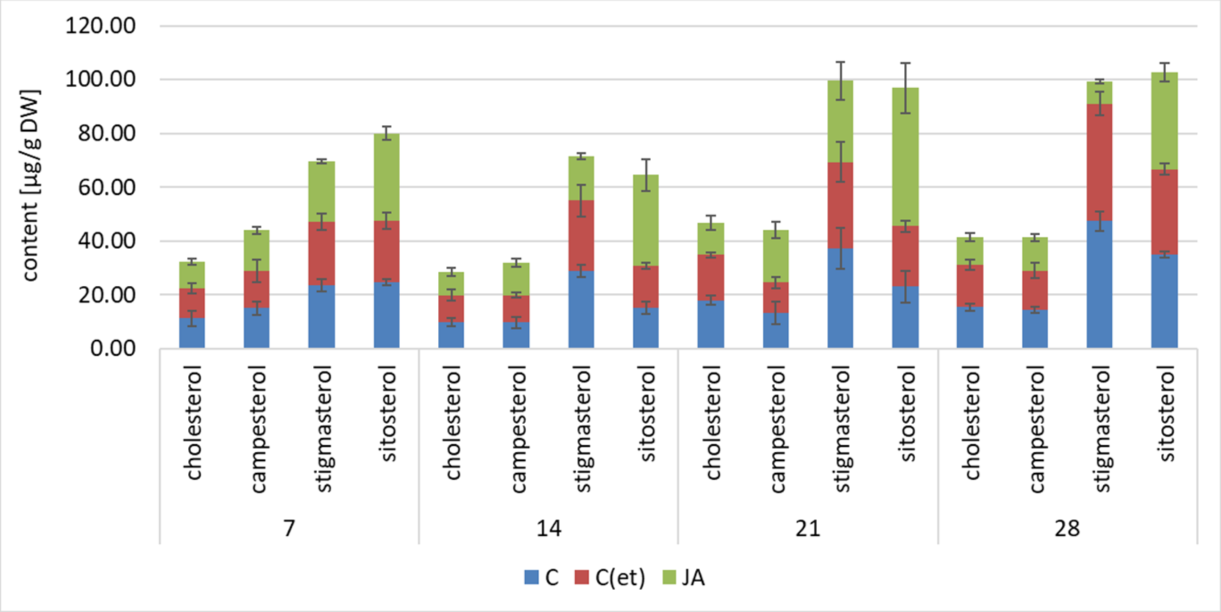

Figure S1. Effect of jasmonic acid elicitation on sterol esters in hairy roots tissue. C- control, C(et)- control samples supplemented with 70% ethanol, JA-jasmonic acid elicited samples. See detailed data and statistical significance in Table S4, Table S6.

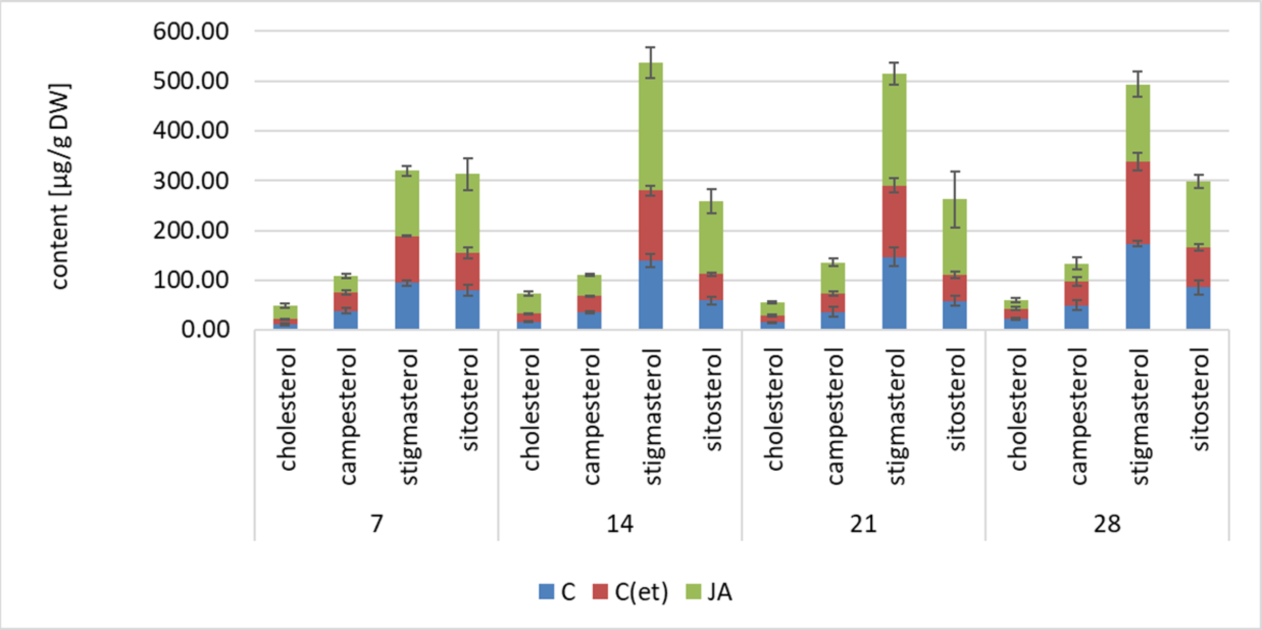

Figure S2. Effect of jasmonic acid elicitation on sterol glycosides in hairy roots tissue. C- control, C(et)- control samples supplemented with 70% ethanol, JA- jasmonic acid elicited samples. See detailed data and statistical significance in Table S5, Table S6.

Table S4. Effect of jasmonic acid elicitation on sterol esters in hairy roots tissue. Data which do not share a common letter are significantly different. Capital letters indicate significant difference in time between plants from the same treatment, lowercase indicate difference between treatments within certain time point.

Compound	Content [μg/g DW± SD]											
	days											
	7			14			21			28		
	C	C(et)	JA	C	C(et)	JA	C	C(et)	JA	C	C(et)	JA
cholesterol	11.22±2.80 BC, a	11.11±1.93 BC, a	10.08±1.17 A, a	9.92±1.58 C, a	9.90±2.04 C, a	8.64±1.52 A, a	17.91±1.66 A, a	16.84±0.80 A, ab	11.91±2.58 A, b	15.32±1.47 AB, ab	15.79±1.81 AB, b	10.12±1.51 A, a
campesterol	15.03±2.51 A, a	13.84±4.21 A, a	15.04±1.44 AB, a	9.62±2.03 A, a	10.15±0.96 A, a	12.18±1.46 B, a	13.22±4.08 A, ab	11.33±2.09 A, b	19.54±3.14 A, a	14.37±1.04 A, a	14.60±2.98 A, a	12.29±1.34 AB, a
stigmasterol	23.49±2.33 C, a	23.61±2.93 B, a	22.49±0.76 AB, a	28.95±2.32 BC, a	25.98±5.85 B, a	16.54±1.18 BC, a	37.13±7.70 AB, a	32.22±7.28 AB, a	30.14±7.13 A, a	47.33±3.73 A, a	43.75±4.54 A, a	8.36±0.77 C, b
sitosterol	24.64±1.18 AB, a	22.96±3.03 AB, a	32.38±2.57 B, a	15.16±2.19 A, a	15.50±1.11 A, a	33.86±5.94 B, b	22.97±5.94 AB, a	22.47±1.99 AB, a	51.38±9.39 A, b	34.75±1.16 B, a	31.93±1.97 B, a	36.09±3.41 B, a
**Total**	**74.37**	**71.52**	**79.99**	**63.65**	**61.52**	**71.22**	**91.22**	**82.85**	**112.96**	**111.77**	**106.06**	**66.87**

Table S5. Effect of jasmonic acid elicitation on sterol glycosides in hairy roots tissue. Data which do not share a common letter are significantly different. Capital letters indicate significant difference in time between plants from the same treatment, lowercase indicate difference between treatments within certain time point.

Compound	Content [μg/g DW± SD]											
	days											
	7			14			21			28		
	C	C(et)	JA	C	C(et)	JA	C	C(et)	JA	C	C(et)	JA
cholesterol	10.86±2.18 A, a	10.40±0.90 A, a	26.81±4.19 B, a	16.39±1.45 A, a	16.13±1.47 A, a	40.32±3.48 A, b	14.53±1.20 A, a	14.26±1.69 A, a	26.62±2.71 B, b	21.43±2.18 B, a	21.44±3.42 B, a	17.74±4.58 C, a
campesterol	38.72±4.73 A, a	36.59±4.81 A, a	32.08±4.51 B, a	35.75±2.73 A, a	32.90±0.97 A, a	41.88±2.48 B, a	36.16±9.64 A, a	36.71±4.33 A, a	62.61±8.02 A, b	49.08±10.27 A, a	48.53±9.02 A, a	35.50±12.13 B, a
stigmasterol	94.03±5.25 B, a	93.99±1.01 A, a	131.68±9.29 A, a	139.14±13.75 AB, a	140.46±9.2 3 AB, a	257.19±30.76 B, b	146.90±19.45 A, a	143.36±14.88 AB, a	224.86±21.70 B, b	173.34±5.36 A, a	164.44±16.9 7 B, a	156.03±25.83 A, a
sitosterol	78.89±10.79 A, a	75.85±10.5 3 A, a	158.40±32.09 A, b	59.02±7.84 A, a	52.67±3.13 A, a	146.12±24.60 A, b	58.17±9.77 A, a	52.50±6.21 A, a	151.73±56.86 A, b	85.42±14.02 A, a	80.26±5.87 A, a	133.17±13.75 A, a
**Total**	**222.50**	**216.84**	**348.97**	**250.30**	**242.15**	**485.52**	**255.76**	**246.82**	**465.83**	**329.27**	**314.66**	**342.44**

Table S6. Analysis of the interaction of treatment and time on sterol esters and sterol glycosides content in hairy roots tissue performed by two-way ANOVA.

	***p* value**			
	cholesterol	campesterol	stigmasterol	sitosterol
**Sterol esters**				
treatment	<0.001	n.s.	<0.001	<0.001
time	<0.001	0.002	<0.001	<0.001
treatment x time	n.s.	0.038	<0.001	<0.001
**Sterol glycosides**				
treatment	<0.001	n.s.	<0.001	<0.001
time	<0.001	0.013	<0.001	n.s.
treatment x time	<0.001	0.001	<0.001	n.s.

TableS7. Effect of jasmonic acid elicitation on neutral terpenoid content in hairy roots tissue. Data which do not share a common letter are significantly different. Capital letters indicate significant difference in time between plants from the same treatment, lowercase indicate difference between treatments within certain time point.

**Compound**	**Content [µg/g DW± SD]**											
	**days**											
	7			14			21			28		
	C	C(et)	JA	C	C(et)	JA	C	C(et)	JA	C	C(et)	JA
β-amyrin	8.11±0.87	7.09±1.71	9.59±0.88	3.85±0.62	4.20±0.34	6.90±1.42	3.78±1.10	3.66±1.51	14.15±1.84	4.17±2.04	4.49±1.41	11.59±2.94
	A, a	A, a	BC, a	A, a	A, a	C, a	A, a	A, a	A, b	A, a	A, a	AB, b
α-amyrin	24.03±2.61	23.87±3.00	13.64±2.42	19.89±2.35	20.78±3.55	27.49±2.26	18.05±1.72	18.61±1.88	30.36±2.55	19.79±5.09	18.88±3.23	20.12±3.28
	A, a	A, a	C, b	A, a	A, a	AB, a	A, a	A, a	A, b	A, a	A, a	BC, a
**Total:**	**32.14**	**30.97**	**23.24**	**23.74**	**24.98**	**34.39**	**21.82**	**22.28**	**44.51**	**23.97**	**23.37**	**31.71**

Table S8. Analysis of the interaction of treatment and time on neutral triterpenoids **(**amyrins) content in hairy roots tissue performed by two-way ANOVA.

	***p* value**	
	β-amyrin	α-amyrin
treatment	<0.001	n.s.
time	0.001	n.s.
treatment x time	<0.001	<0.001

Table S9. Effect of jasmonic acid elicitation on free oleanolic acid (OA) content in hairy roots tissue. Data which do not share a common letter are significantly different. Capital letters indicate significant difference in time between plants from the same treatment, lowercase indicate difference between treatments within certain time point.

**Compound**	**Content [µg/g DW± SD]**											
	**days**											
	7			14			21			28		
	C	C(et)	JA	C	C(et)	JA	C	C(et)	JA	C	C(et)	JA
OA	12.26±1.19	11.51±2.38	233.87±7.21	12.57±3.15	12.77±2.60	258.23±83.44	24.09±2.32	24.63±3.19	409.93±108.24	27.14±6.32	29.09±3.98	476.19±94.55
	A, a	A, a	A, b	A, a	A, a	A, b	A, a	A, a	B, b	A, a	A, a	B, b

Table S10. Analysis of the interaction of treatment and time on free oleanolic acid (OA) content in hairy roots tissue performed by two-way ANOVA.

	***p* value**
	OA
treatment	<0.001
time	0.001
treatment x time	0.002

Table S11. Effect of jasmonic acid elicitation on oleanolic acid saponins (OA) content in hairy roots tissue. Data which do not share a common letter are significantly different. Capital letters indicate significant difference in time between plants from the same treatment, lowercase indicate difference between treatments within certain time point.

**Compound**	**Content [μg/g DW± SD]**											
	**days**											
	7			14			21			28		
	C	C(et)	JA	C	C(et)	JA	C	C(et)	JA	C	C(et)	JA
OA	1583.56± 171.84 A, a	1604.97± 180.11 A, a	123998.36± 10694.23 A, b	2223.25± 339.69 A, a	1984.91± 273.63 A, a	104230.59± 2204.90 A, b	2480.48± 298.24 A, a	2388.99± 472.87 A, a	159514.22± 30538.66 B, b	1918.20± 626.65 A, a	1853.05± 380.85 A, a	164286.71± 15220.88 B, b

Table S12. Analysis of the interaction of treatment and time on oleanolic acid (OA) saponins content in hairy roots tissue performed by two-way ANOVA.

	***p* value**
	OA
treatment	<0.001
time	0.001
treatment x time	<0.001

Table S13. Effect of jasmonic acid elicitation on oleanolic acid saponins (OA) released to the culture medium. Data which do not share a common letter are significantly different. Capital letters indicate significant difference in time between plants from the same treatment, lowercase indicate difference between treatments within certain time point.

**Compound**	**Content [mg/L•g DW± SD]**											
	**days**											
	7			14			21			28		
	C	C(et)	JA	C	C(et)	JA	C	C(et)	JA	C	C(et)	JA
OA	0.21±0.08 A, a	0.20±0.04 A, a	19.08±1.88 A, a	0.30±0.10 A, a	0.27±0.05 A, a	161.12±10.61 B, b	0.45±0.24 A, a	0.35±0.21 A, a	202.50±34.57 BC, b	6.54±1.92 A, a	5.67±1.26 A, a	218.59±33.84 C, b

Table S14. Analysis of the interaction of treatment and time on oleanolic acid (OA) saponins released to the medium performed by two-way ANOVA.

	***p* value**
	OA
treatment	<0.001
time	<0.001
treatment x time	<0.001

Table S15. Content of free sterols, neutral triterpenoids and triterpenoid acids in *C. officinalis* roots. Data which do not share a common letter are significantly different. Capital letters indicate significant difference in time between plants from the same treatment, lowercase indicate difference between treatments within certain time point.

**Compound**	**Content [μg/g DW± SD]**					
	**Days**					
	7			14		
	C	C(et)	JA	C	C(et)	JA
**Free sterols:**						
cholesterol	11.32±0.55 A, a	5.08±0.64 A, b	8.64±2.11 A, a	6.41±0.83 B, a	4.23±1.23 A, a	7.39±0.85 A, a
campesterol	84.75±6.91 A, a	81.14±13.31 A, a	73.62±14.29 A, a	50.89±2.41 B, a	38.23±6.42 B, a	35.51±1.96 B, a
stigmasterol	804.98±27.26 A, a	646.12±55.14 A, b	539.31±56.37 A, b	433.79±50.21 B, a	366.29±55.17 B, a	280.13±9.75 B, b
sitosterol	446.97±41.21 A, a	349.19±63.60 A, a	350.67±21.85 A, a	316.98±24.32 B, a	239.74±24.88 B, a	210.28±27.55 B, b
sitostanol	122.45±12.46 A, a	117.33±19.83 A, a	73.89±24.85 A, b	62.63±7.26 B, a	62.85±4.77 B, a	52.22±8.22 A, a
tremulone	32.16±2.72 A, a	10.54±3.22 A, b	47.53±4.93 A, c	10.50±0.82 B, a	10.93±1.52 A, a	28.30±5.00 B, b
sitostenone	115.82±9.30 A, a	118.71±9.93 A, a	112.32±10.36 A, a	48.34±12.21 B, a	46.33±5.87 B, a	31.68±4.69 B, a
cycloartenol acetate	21.46±1.65 A, a	23.76±2.28 A, a	21.02±1.36 A, a	14.79±1.21 B, a	9.77±1.49 B, b	8.63±2.01 B, b
stigmastan-3,6-dione	n.d. A, a	16.16±2.05 A, b	12.55±2.02 A, b	11.15±2.75 B, a	13.03±2.17 A, a	9.94±1.13 A, a
**Total sterols:**	**1639.90**	**1368.04**	**1239.55**	**955.49**	**791.40**	**664.07**
**Neutral triterpenoids:**						
β-amyrin	47.52±4.89 A, a	46.40±12.13 A, a	74.18±12.22 A, b	31.70±1.93 A, a	29.96±5.24 A, a	48.11±8.83 B, a
α-amyrin	38.56±7.91 A, a	34.17±6.10 A, a	54.36±9.23 A, a	30.81±2.02 A, a	28.61±6.71 A, a	36.95±12.57 A, a
**Sum of amyrins:**	**86.08**	**80.57**	**128.54**	**62.52**	**58.57**	**85.06**
fridelinol	77.05±7.70 A, a	87.16±22.64 A, a	112.40±14.52 A, a	80.42±16.54 A, a	78.47±15.07 A, a	107.09±9.23 A, a
friedelin	44.76±4.88 A, a	45.49±8.05 A, a	72.26±7.86 A, b	49.37±3.34 A, a	49.89±5.28 A, a	64.89±10.77 A, a
**Sum of friedooleanans:**	**121.81**	**132.66**	**184.66**	**129.79**	**128.36**	**171.98**
**Triterpenoid acids:**						
OA	19.80±0.88 A, ab	18.01±1.62 A, b	24.17±1.84 A, a	23.45±2.67 A, a	17.96±1.47 A, a	31.86±3.03 B, b
UA	32.92±3.98 A, a	33.42±5.68 A, a	42.93±6.26 A, a	44.84±4.81 A, a	45.77±5.27 A, a	52.76±3.30 A, a
**Sum of triterpenoid acids:**	**52.72**	**51.43**	**67.10**	**68.29**	**63.73**	**84.62**

Table S16. Analysis of the interaction of treatment and time on free sterols in *C. officinalis* roots performed by two-way ANOVA.

	***p* value**							
	cholesterol	campesterol	stigmasterol	sitosterol	sitostanol	tremulone	cycloartenol acetate	stigmastan-3,6-dione
treatment	<0.001	n.s.	<0.001	0.001	0.008	<0.001	0.019	<0.001
time	0.001	<0.001	<0.001	<0.001	<0.001	<0.001	<0.001	n.s.
treatment x time	0.019	n.s.	n.s.	n.s.	n.s.	<0.001	0.007	<0.001

Table S17. Analysis of the interaction of treatment and time on neutral triterpenoids (amyrins and friedooleanans) in *C. officinalis* roots performed by two-way ANOVA.

	***p* value**			
	β-amyrin	α-amyrin	fridelinol	friedelin
treatment	0.001	0.025	0.008	<0.001
time	<0.001	0.020	n.s.	n.s.
treatment x time	n.s.	n.s.	n.s.	n.s.

Table S18. Analysis of the interaction of treatment and time on triterpenoid acids in *C. officinalis* roots performed by two-way ANOVA.

	***p* value**	
	OA	UA
treatment	<0.001	0.016
time	0.002	<0.001
treatment x time	0.022	n.s.

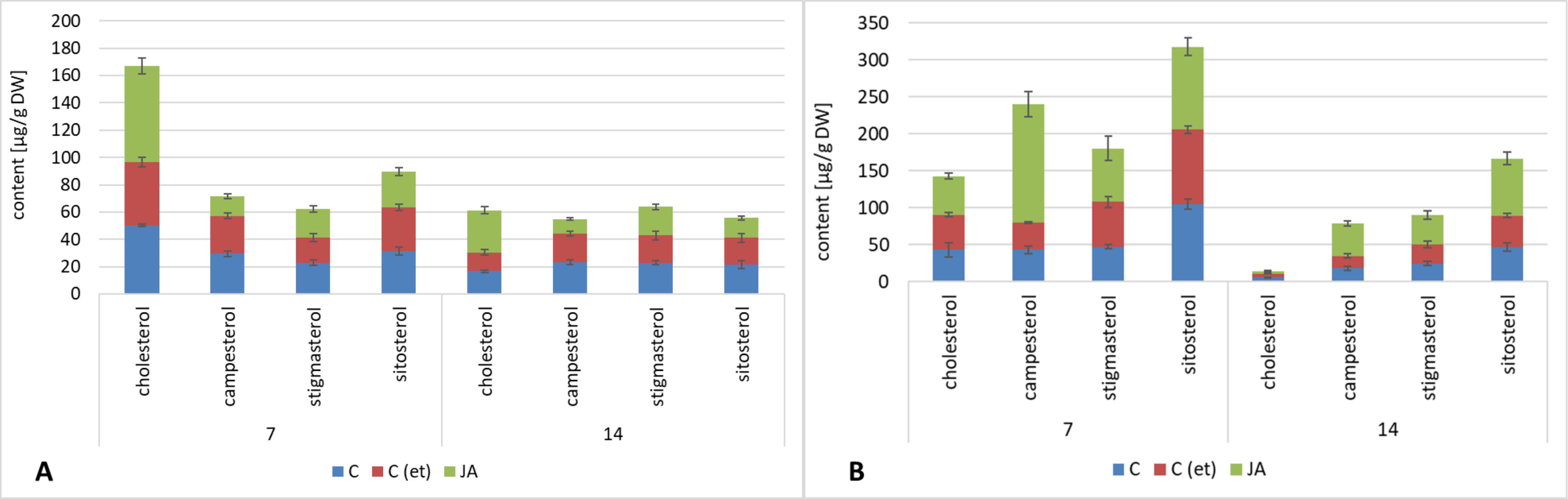

Figure S3. Effect of jasmonic acid elicitation on sterol esters (A) and sterol glycosides (B) in *C. officinalis* roots. C- control, C(et)- control samples supplemented with 70% ethanol, JA- jasmonic acid elicited samples. See detailed data and statistical significance in Tables S19 and S20.

Table S19. Content of sterols conjugated in sterol esters and sterol glycosides in *C. officinalis* roots. Data which do not share a common letter are significantly different. Capital letters indicate significant difference in time between plants from the same treatment, lowercase indicate difference between treatments within certain time point.

**Compound**	**Content [µg/g DW± SD]**					
	**Days**					
	7			14		
	C	C(et)	JA	C	C(et)	JA
**Sterol esters:**						
cholesterol	50.28±1.01 A, a	46.21±3.54 A, a	70.65±5.84 A, b	16.65±0.91 B, a	13.81±2.05 B, a	30.69±2.61 B, b
campesterol	29.45±2.28 A, a	27.84±1.95 A, a	14.34±1.95 A, b	23.35±1.51 B, a	21.05±1.70 B, a	10.56±0.80 A, b
stigmasterol	22.98±2.09 A, a	18.24±2.91 A, a	21.20±2.23 A, a	22.83±1.51 A, a	19.99±3.20 A, a	21.17±2.00 A, a
sitosterol	31.49±3.02 A, a	31.85±2.37 A, a	26.38±2.76 A, a	21.43±2.92 A, a	19.75±3.28 A, a	14.61±1.53 B, a
**Total:**	**134.21**	**124.14**	**132.57**	**84.27**	**74.60**	**77.03**
**Sterol glycosides:**						
cholesterol	42.87±9.54 A, a	47.68±2.75 A, a	52.15±4.29 A, a	5.59±0.37 B, a	5.23±0.72 B, a	3.35±0.54 B, a
campesterol	43.33±5.07 A, a	37.00±1.11 A, a	159.47±16.68 A, b	18.41±2.86 B, a	16.01±2.91 B, a	44.16±3.42 B, b
stigmasterol	47.29±2.84 A, a	60.57±7.40 A, ab	72.28±16.33 A, b	24.52±2.70 B, a	25.48±4.58 B, a	39.74±5.46 B, a
sitosterol	104.69±6.39 A, a	101.03±5.17 A, a	112.05±11.73 A, a	46.73±5.23 B, a	42.15±2.85 B, a	77.77±8.24 B, b
**Total:**	**238.19**	**246.28**	**395.95**	**95.25**	**88.87**	**165.03**

Table S20. Analysis of the interaction of treatment and time on sterol esters and sterol glycosides in *C. officinalis* roots performed by two-way ANOVA.

	***p* value**			
	cholesterol	campesterol	stigmasterol	sitosterol
**Sterol esters**				
treatment	<0.001	<0.001	n.s.	0.005
time	<0.001	<0.001	n.s.	<0.001
treatment x time	n.s.	n.s.	n.s.	n.s.
**Sterol glycosides**				
treatment	n.s.	<0.001	0.003	<0.001
time	<0.001	<0.001	<0.001	<0.001
treatment x time	n.s.	<0.001	n.s.	0.018

Table S21. Content of free sterols, neutral triterpenoids and triterpenoid acids in *C. officinalis* shoots. Data which do not share a common letter are significantly different. Capital letters indicate significant difference in time between plants from the same treatment, lowercase indicate difference between treatments within certain time point.

Compound	Content [μg/g DW± SD]					
	Days					
	7			14		
	C	C(et)	JA	C	C(et)	JA
**Free sterols:**						
cholesterol	9.15±1.48 A, a	8.25±1.00 A, a	8.31±3.16 A, a	16.83±3.54 B, a	5.49±1.27 A, b	2.88±0.51 A, b
campesterol	26.07±4.56 A, a	25.72±1.61 A, a	25.35±4.77 A, a	43.66±0.67 B, a	24.00±2.57 A, b	13.40±0.70 B, c
stigmasterol	385.75±82.44 A, a	441.33±18.62 A, a	455.50±80.28 A, a	390.37±32.24 A, a	397.08±38.47 A, a	276.02±8.22 B, a
sitosterol	215.58±29.77 A, a	209.87±12.89 A, a	183.17±26.28 A, a	154.06±9.90 B, a	200.31±22.40 A, a	94.05±2.65 B, b
sitostanol	16.87±2.69 A, a	11.11±0.61 A, a	13.38±3.04 A, a	19.85±6.79 A, a	16.88±2.87 A, a	13.23±1.88 A, a
tremulone	6.05±1.64 A, a	10.02±2.21 A, a	42.04±2.29 A, b	7.97±2.94 A, a	8.84±1.88 A, a	14.04±3.39 B, a
sitostenone	13.48±4.45 A, a	18.09±4.25 A, a	19.37±1.36 A, a	13.47±2.16 A, a	12.70±3.02 A, a	10.26±1.67 B, a
**Total sterols:**	**672.96**	**724.39**	**747.12**	**646.21**	**665.31**	**423.89**
**Neutral triterpenoids:**						
β-amyrin	39.83±2.81 A, a	44.84±4.66 A, a	39.78±5.49 A, a	38.11±4.15 A, a	43.41±9.36 A, a	29.99±3.05 A, a
α-amyrin	50.35±6.97 A, a	51.61±3.91 A, a	55.43±4.75 A, a	53.03±6.74 A, a	62.30±3.76 A, a	67.24±7.55 A, a
**Sum of amyrins:**	**90.18**	**96.46**	**95.21**	**91.14**	**105.71**	**97.23**
**Triterpenoid acids:**						
OA	8.11±1.46 A, a	2.96±0.85 A, b	9.49±2.63 A, a	16.34±1.04 B, a	12.33±0.95 B, a	10.39±2.53 A, b
UA	24.72±2.55 A, a	12.54±2.59 A, b	10.87±3.15 A, b	19.81±1.48 A, a	9.21±1.31 A, b	4.87±2.38 A, b
**Sum of triterpenoid acids:**	**32.82**	**15.50**	**20.36**	**36.15**	**21.54**	**15.25**

Table S22. Analysis of the interaction of treatment and time on free sterols in *C. officinalis* shoots performed by two-way ANOVA.

	***p* value**						
	cholesterol	campesterol	stigmasterol	sitosterol	sitostanol	tremulone	sitostenone
treatment	<0.001	<0.001	n.s.	<0.001	n.s.	<0.001	n.s.
time	n.s.	n.s.	0.011	<0.001	n.s.	<0.001	0.006
treatment x time	<0.001	<0.001	0.025	0.014	n.s.	<0.001	n.s.

Table S23. Analysis of the interaction of treatment and time on neutral triterpenoids (amyrins) in *C. officinalis* shoots performed by two-way ANOVA.

	***p* value**	
	β-amyrin	α-amyrin
treatment	0.036	0.043
time	n.s.	0.010
treatment x time	n.s.	n.s.

Table S24. Analysis of the interaction of treatment and time on triterpenoid acids in *C. officinalis* shoots performed by two-way ANOVA.

	***p* value**	
	OA	UA
treatment	0.002	<0.001
time	<0.001	0.001
treatment x time	0.002	n.s.

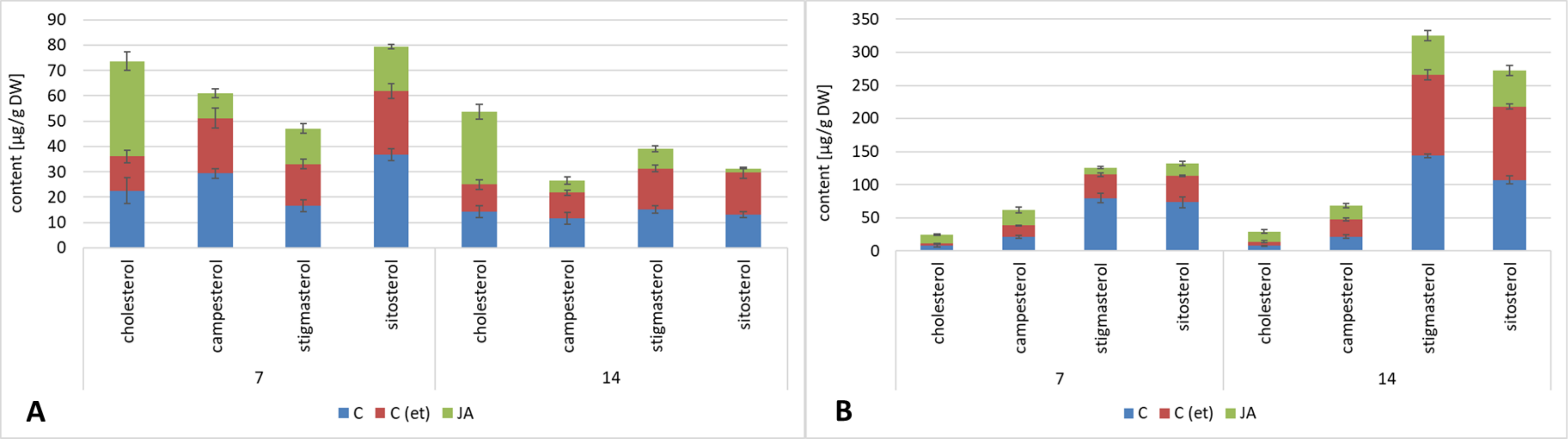

Figure S4. Effect of jasmonic acid elicitation on sterol esters (A) and sterol glycosides (B) in *C. officinalis* shoots. C- control, C(et)- control samples supplemented with 70% ethanol, JA- jasmonic acid elicited samples. See detailed data and statistical significance in Tables S25 and S26.

Table S25. Content of sterols conjugated in sterol esters and sterol glycosides in *C. officinalis* shoots. Data which do not share a common letter are significantly different. Capital letters indicate significant difference in time between plants from the same treatment, lowercase indicate difference between treatments within certain time point.

Compound	Content [µg/g DW± SD]					
	Days					
	7			14		
	C	C(et)	JA	C	C(et)	JA
**Sterol esters:**						
cholesterol	22.57±4.99 A, a	13.47±2.59 A, b	37.57±3.66 A, c	14.26±2.43 A, a	10.67±1.94 A, a	28.87±2.89 A, b
campesterol	29.35±1.90 A, a	21.82±3.96 A, b	9.75±1.78 A, c	11.65±2.29 B, a	10.12±1.00 B, ab	4.74±1.55 A, b
stigmasterol	16.67±2.37 A, a	16.39±1.97 A, a	14.03±1.92 A, a	15.17±1.37 A, a	16.06±1.31 A, a	7.94±1.13 B, b
sitosterol	36.73±2.33 A, a	25.18±2.93 A, b	17.40±0.89 A, c	12.99±1.16 B, a	16.67±2.16 B, a	1.67±0.08 B, b
**Total:**	**105.31**	**76.85**	**78.75**	**54.07**	**53.52**	**43.22**
**Sterol glycosides:**						
cholesterol	7.65±2.29 A, a	3.08±0.45 A, b	13.61±1.07 A, c	7.77±0.46 A, a	5.91±1.55 A, a	15.30±2.68 A, b
campesterol	21.22±2.38 A, a	17.01±0.45 A, a	23.36±4.75 A, a	21.47±2.87 A, a	26.11±2.06 B, a	20.27±3.29 A, a
stigmasterol	79.70±6.78 A, a	35.60±2.65 A, b	10.47±1.85 A, c	143.77±2.95 B, a	122.36±8.00 B, b	59.09±7.24 B, c
sitosterol	73.29±8.55 A, a	40.30±0.86 A, b	18.48±3.29 A, c	107.17±6.18 B, a	110.82±3.68 B, a	54.24±7.50 B, b
**Total:**	**181.86**	**96.00**	**65.92**	**280.18**	**265.19**	**148.91**

Table S26. Analysis of the interaction of treatment and time on sterol esters and sterol glycosides in *C. officinalis* shoots performed by two-way ANOVA.

	***p* value**			
	cholesterol	campesterol	stigmasterol	sitosterol
**Sterol esters**				
treatment	<0.001	<0.001	<0.001	<0.001
time	0.001	<0.001	0.007	<0.001
treatment x time	n.s.	0.002	0.033	<0.001
**Sterol glycosides**				
treatment	<0.001	n.s.	<0.001	<0.001
time	n.s.	n.s.	<0.001	<0.001
treatment x time	n.s.	0.010	<0.001	<0.001

Table S27. Content of oleanolic acid saponins (OA) in roots and shoots of *C. officinalis*. Data which do not share a common letter are significantly different. Capital letters indicate significant difference in time between plants from the same treatment, lowercase indicate difference between treatments within certain time point.

**Organ**	**Content [µg/g DW± SD]**					
	**Days**					
	7			14		
	C	C(et)	JA	C	C(et)	JA
**Roots**						
OA	2474.64±205.76 A, a	1637.07±141.39 A, a	3274.73±89.53 A, a	4233.38±298.04 A, a	7414.07±433.90 B, b	9564.53±2158.24 B, b
**Shoots**						
OA	11588.57±855.98 A, a	11531.16±2252.04 A, a	22000.80±2718.21 A, b	18818.27±360.98 B, a	15627.33±1849.59 A, a	31298.40±385.69 B, b

Table S28. Analysis of the interaction of treatment and time on oleanolic acid (OA) saponins in roots and shoots of *C. officinalis* performed by two-way ANOVA.

	***p* value**	
	OA roots	OA shoots
treatment	<0.001	<0.001
time	<0.001	<0.001
treatment x time	0.002	n.s.

Table S29. Basic physical and chemical charactrerization of Universal soil „Athena" including: pH, salinity, concentration of nitrogen (N), potassium oxide (K_2O) and phosphates (P_2O_5).

Parameters	Units	„Athena" soil
pH	-	5.5-6.5
Salinity	g/L	1.5
N	mg/L	180
K_2O	mg/L	220
P_2O_5	mg/L	160
